# *Streptomyces* as Microbial Chassis for Heterologous Protein Expression

**DOI:** 10.3389/fbioe.2021.804295

**Published:** 2021-12-21

**Authors:** Soonkyu Hwang, Yongjae Lee, Ji Hun Kim, Gahyeon Kim, Hyeseong Kim, Woori Kim, Suhyung Cho, Bernhard O. Palsson, Byung-Kwan Cho

**Affiliations:** ^1^ Department of Biological Sciences, Korea Advanced Institute of Science and Technology, Daejeon, South Korea; ^2^ KAIST Institute for the BioCentury, Korea Advanced Institute of Science and Technology, Daejeon, South Korea; ^3^ Department of Bioengineering, University of California, San Diego, La Jolla, CA, United States; ^4^ Department of Pediatrics, University of California, San Diego, La Jolla, CA, United States; ^5^ Novo Nordisk Foundation Center for Biosustainability, Technical University of Denmark, Lyngby, Denmark; ^6^ Innovative Biomaterials Research Center, KAIST Institutes, Korea Advanced Institute of Science and Technology, Daejeon, South Korea

**Keywords:** *streptomyces*, heterologous expression, chassis, recombinant protein, secondary metabolite

## Abstract

Heterologous production of recombinant proteins is gaining increasing interest in biotechnology with respect to productivity, scalability, and wide applicability. The members of genus *Streptomyces* have been proposed as remarkable hosts for heterologous production due to their versatile nature of expressing various secondary metabolite biosynthetic gene clusters and secretory enzymes. However, there are several issues that limit their use, including low yield, difficulty in genetic manipulation, and their complex cellular features. In this review, we summarize rational engineering approaches to optimizing the heterologous production of secondary metabolites and recombinant proteins in *Streptomyces* species in terms of genetic tool development and chassis construction. Further perspectives on the development of optimal *Streptomyces* chassis by the design-build-test-learn cycle in systems are suggested, which may increase the availability of secondary metabolites and recombinant proteins.

## Introduction

Many efforts have been made to produce recombinant proteins on a large industrial scale. Heterologous protein expression in the platform host by the introduction of the gene of interest is the most promising approach in several aspects. Heterologous expression hosts can overcome the complexities associated with native hosts, such as slow and fastidious growth, limited molecular biology tools, scarce genetic information, and low productivity (Park et al., 2020). Several representative heterologous expression hosts have been used according to their specialized characteristics and product types (Huo et al., 2019; Zhang et al., 2019; Kang and Kim, 2021; Pham et al., 2021). For example, *Escherichia coli* is the most extensively studied and used for heterologous protein expression, as it exhibits rapid growth, ease of genetic manipulation, and high productivity. *Bacillus subtilis* is another extensively used Gram-positive bacteria that acts as an efficient workhorse for the production of industrial enzymes and pharmaceuticals, having robust growth and good genetic tractability, along with many endogenous proteases. Beyond the microbial hosts, *Saccharomyces cerevisiae* is a commonly used eukaryotic host for recombinant protein production due to its recombinant DNA stability, easy genome engineering strategies, and the ability to provide post-translational modifications. Recently, a high-throughput platform for heterologous protein expression for fungal biosynthetic gene clusters (BGCs) in *S. cerevisiae*, named HEx, was established to successfully produce diverse fungal secondary metabolites (SMs) (Harvey et al., 2018). Chinese hamster ovary (CHO) cells are the mammalian cell line that dominates the other recombinant protein production hosts because of their capacity to express large and complex recombinant proteins, but genetic engineering and clonal selection are more difficult than microbial hosts.

Among the potential microbial chassis, the genus *Streptomyces,* a soil-derived Gram-positive bacterium with a high GC content genome, is an attractive microbial host for heterologous protein expression (Liu et al., 2018a). To survive under limited nutrient conditions and compete with various other microorganisms in soil, *Streptomyces* has a complex developmental cycle that sporulates after mycelial growth and produces diverse SMs (Van Wezel and Mcdowall, 2011; Barbuto Ferraiuolo et al., 2021). In addition, various enzymes are known to be produced and secreted to degrade complex nutrients, such as lignocellulose, and to perform various reactions on different substrates (Berini et al., 2020). SMs are synthesized by a series of reactions catalyzed by the biosynthetic enzymes encoded in the BGCs. For example, polyketide synthases (PKSs) and non-ribosomal peptide synthetases (NRPSs) are organized by multiple domains, and even by multiple modules in several types that constitute a large biosynthetic machinery (Hwang et al., 2020). In this sense, *Streptomyces* are likely to be more amenable to producing functional biosynthetic enzymes from BGCs relative to other microorganisms, such as *E. coli*. Although heterologous expression of BGC enzymes has been successful in *Streptomyces* platform hosts, several inadequacies and overcoming strategies have been identified.

In this review, the advantages and limitations of *Streptomyces* as a chassis for heterologous protein expression are discussed. Then, BGCs and recombinant protein examples of heterologous expression in *Streptomyces* are reviewed, particularly focusing on genetic tool development and chassis construction. In addition, future perspectives on effective strategies with respect to the design-build-test-learn (DBTL) cycle in synthetic biology are also proposed.

## *Streptomyces* as the Heterologous Protein Expression Host: Advantages and Limitations

### *Streptomyces* as a Heterologous Protein Expression Host

The growth of several *Streptomyces* species is robust and scalable, as they have been intensively used for the industrial production of SMs (Berini et al., 2020). They are also favorable hosts for heterologous production of recombinant proteins. This is mainly due to their protein secretion systems in the extracellular milieu (Liu et al., 2013; Hamed et al., 2018; Berini et al., 2020). Secretion is beneficial in terms of protein folding, because the extracellular medium is an ordinarily oxidizing environment that promotes disulfide bond formation for correct folding, while the cytoplasm is a reducing environment that impedes the disulfide bonds (Anne et al., 2014). *Streptomyces* is a Gram-positive bacterium that has a cell wall layer without an outer membrane, and its secretion into the extracellular space is easier than that of Gram-negative bacteria. As the cell disruption is not required, the downstream protein purification from secreted proteins by *Streptomyces* is more comfortable without contamination by intracellular proteins (Sommer et al., 2009). In addition, *Streptomyces* exhibits low toxicity and does not contain lipopolysaccharides (LPSs), which may act as potent immunostimulatory endotoxins (Berini et al., 2020), allowing easy downstream purification processes. Moreover, *S. lividans*, the most frequently used *Streptomyces* host for recombinant protein production, is known to have low restriction enzyme and proteolytic activities, enabling efficient introduction of the recombinant DNA and high yield of the protein, respectively (Anne et al., 2014).

### *Streptomyces* as a Heterologous Expression Host of Secondary Metabolite BGCs

The number of BGCs per *Streptomyces* genome was predicted to be 36.5 on average, by antiSMASH, reflecting the potential diversity of SMs and their biosynthetic enzymes (Lee et al., 2021). As these biosynthetic enzymes have specific and sophisticated structures, the cellular environment in the heterologous expression host to maintain their structures is important for their function (Hwang et al., 2020). Therefore, *Streptomyces* heterologous expression hosts are favorable for producing functional biosynthetic enzymes encoded in the BGCs of *Streptomyces* sources relative to other microorganisms, such as *E. coli*.

Correct folding is closely correlated with enzyme solubility. In particular, correct incorporation of multiple domains and modules is necessary for enzyme solubility. For example, exchanging the dehydratase domain or linker domain for combinatorial biosynthesis of polyketides (PKs) often generates insoluble aggregates of enzymes (Liew et al., 2012; Cai and Zhang, 2018). In addition, uncoupling transcription and translation may lead to insolubility due to misfolding, which requires optimization of the transcription and translation rate for heterologous expression (Skiba et al., 2018). Codon usage has a significant impact on the translation rate of GC-rich BGC sequences that are likely to be translated inefficiently at low-use codons in *E. coli* (Ke and Yoshikuni, 2020)*.* However, *Streptomyces* hosts do not need additional codon optimization to improve the translation efficiency for BGC expression. Moreover, the cytoplasmic redox state of the heterologous expression host should be similar to that of the native host to stimulate correct disulfide bonds. For example, a large size of the main synthetase of the glycopeptide NRPS chloroeremomycin BGC CepA was expressed in *E. coli*, but it showed low activity, perhaps due to incorrect folding by the non-optimal redox state of *E. coli* (Trauger and Walsh, 2000; Skiba et al., 2018)*.* Therefore, *Streptomyces* is a more convenient host for the expression of functional large BGC enzymes. Chaperone is also essential for the functioning of biosynthesis enzymes in several examples, including MbtH homologs within several NRPS BGCs and PqqD family chaperones within lasso peptide BGCs (Shao et al., 2019). Other considerations for the heterologous expression of functional enzymes, including cDNA selection, expression system optimization, tagging, pH, temperature, and cofactor, were reviewed in a previous study (Skiba et al., 2018).

Metabolic background of *Streptomyces* plays a key role on successful heterologous production of SMs, especially in terms of substrate availability. As *Streptomyces* hosts produce a variety of SMs from native BGCs encoded in their genomes, they naturally possess diverse reactions to produce sufficient precursors such as propionyl-CoA, methylmalonyl-CoA, benzoyl-CoA, and others for PKs, and p-aminobenzoic acid, 3-amino-5-hydroxybenzoic acid, and various *β*-amino acids for NRPs, respectively (Pfeifer and Khosla, 2001; Luo et al., 2016; Sharma et al., 2021). Despite of the ability, sufficient precursor supply should be additionally optimized for the high yield by preventing the undesired flux to other pathways competing for the same precursor molecules to enhance the productivity.

Next, SM biosynthesis usually involves many diverse post-modifications at the final step by tailoring enzymes governing phosphorylation, methylation, acetylation, cyclization, farnesylation, and glycosylation (Pfeifer and Khosla, 2001; Liu et al., 2018a). As *Streptomyces* heterologous expression hosts have a high possibility of expressing functional tailoring enzymes, they would be better than other hosts. However, tailoring enzymes are distinct even between *Streptomyces* species that are carefully chosen for co-expression. For instance, a farnesyl transferase-coding gene was not found in the genome of *S. albus*, indicating the requirement for additional introduction of this enzyme to produce related SMs (Liu et al., 2018a). Lastly, high production of SMs, especially antibiotics, requires the transport and resistance genes to have self-tolerance against the products. *Streptomyces* heterologous expression hosts may have native transporters or resistance proteins with broad substrate specificity to have higher self-tolerance than other heterologous hosts. With the availability of precursors and the presence of other additional genes, such as regulatory, tailoring, transport, and resistance genes, *Streptomyces* heterologous expression hosts are a good choice because they are not very different from the native BGC hosts as compared to *E. coli*.

**FIGURE 1:**
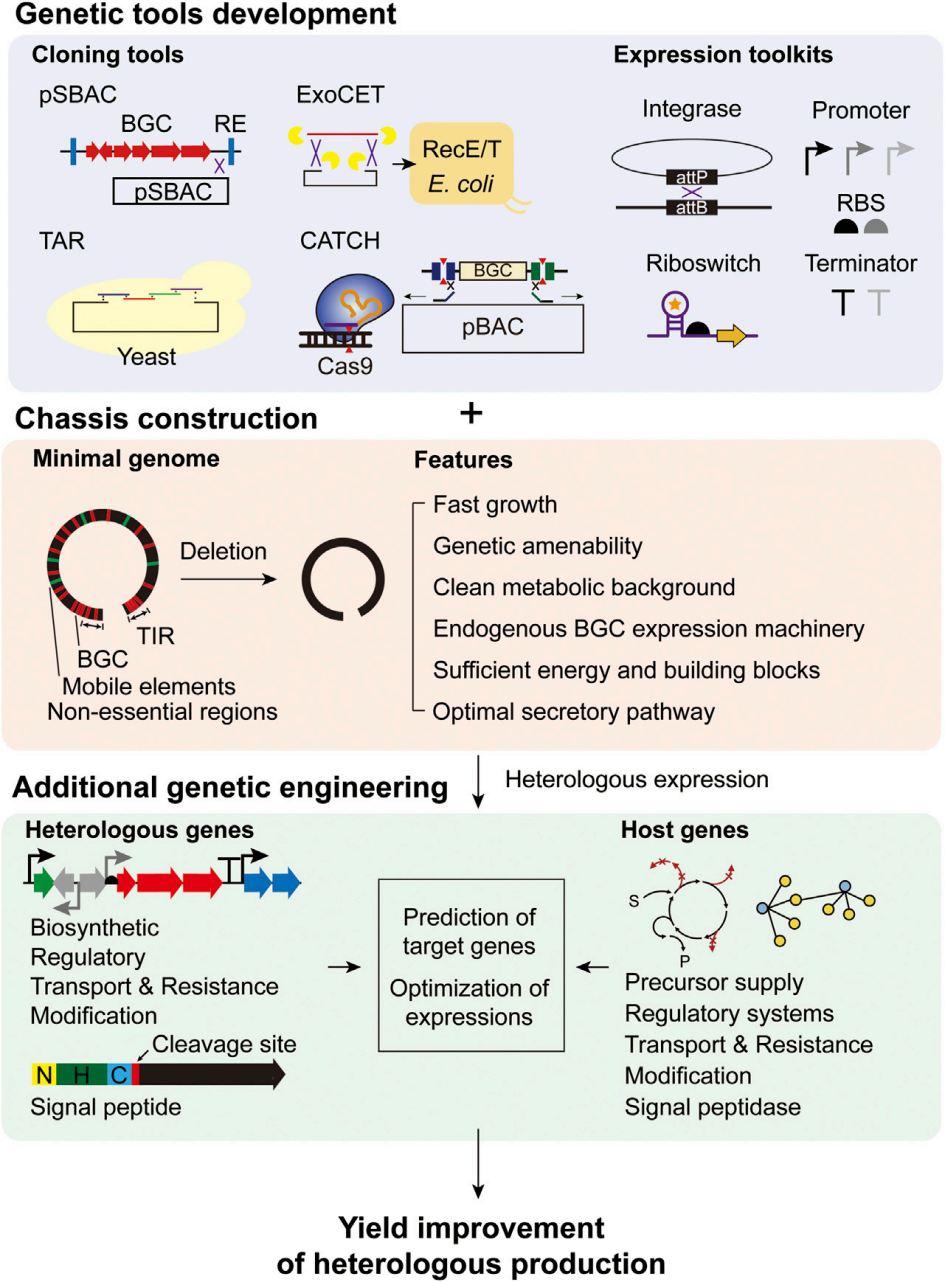
Rational engineering strategies for heterologous production of secondary metabolites and recombinant proteins in *Streptomyces*.

In summary, *Streptomyces* is a specialized host for BGC expression due to its functional biosynthetic enzyme expression, substrate availability, and presence of other accessory genes. Recombinant proteins are also effectively expressed in *Streptomyces*, mainly due to their secretion systems. However, there are also some drawbacks compared to other heterologous hosts in terms of the robustness of growth, genetic tools, and genetic information. To overcome these limitations, rational engineering approaches for heterologous protein production have been developed for *Streptomyces*, as presented in the following sections (Figure 1).

## Genetic Tools for Rational Engineering of BGC Heterologous Expression in *Streptomyces*

Since a number of genes are included in a gene cluster, BGCs are generally large, ranging from 10 kb to over 200 kb in size. Cloning of large BGCs requires considerable time and effort and is limited in terms of efficiency. Therefore, many tools have been developed to improve the efficiency and fidelity of *Streptomyces* heterologous hosts. This section focuses on the cloning or assembly strategies that have been developed so far. The following section will provide the steps to improve heterologous protein expression via the engineering of genetic elements.

### Strategies for the Cloning and Assembly of BGCs

The traditional cloning strategy is a library-based method in which genomic DNA is digested with restriction enzymes and ligated into vectors, such as cosmids, P1-derived atomic chromosome (PAC), and the bacterial atomic chromosome (BAC). Cosmid is a vector based on the *E. coli* λ bacteriophage, that can accommodate a small BGC of up to 42 kb (Jones et al., 2013), while BAC containing an F-factor or PAC vector can accommodate a relatively large BGC, up to 490 kb in BAC (Zimmer and Verrinder Gibbins, 1997) and 300 kb in PAC (Tu et al., 2018). The library-based cloning method was problematic in that it required numerous clones to be screened by polymerase chain reaction (PCR). Instead, direct cloning strategies for BGCs without library construction have emerged. Direct cloning strategies were further divided depending on whether the cloning was performed *in vivo* or *in vitro* (Table 1).

**TABLE 1:** Selected examples of strategies for cloning and assembly of secondary metabolite BGCs.

Strategies	Principle	NP (class, size (kb))	Heterologous host	Advantage	Limitation	Reference
Genomic library	• Restriction digestion and ligation	A54145 (NRPS, 60)	*S. ambofaciens* BES2074	• Suitable for small to large size of fragments	• Efficiency of ligation and transformation is low	(Alexander et al., 2010; Li et al., 2017b; Tan et al., 2017; D’agostino and Gulder, 2018; Liu et al., 2018b; Tu et al., 2018)
	• Integrative into chromosome with prophage cassette	Kinamycin (PKS, >63)	*S. albus* J1074			
		Abyssomicin (PK, 74)	*S. coelicolor* M1152	• Genome sequence data is not required	• Screening colonies could be time consuming and laborious process	
		Spinosad (PK, 80)	*S. albus* J1074			
			*S. lividans* TK24			
		FK506 (PKS, 120)	*S. coelicolor* strains			
pSBAC	• Restriction digestion and ligation	Pikromycin (PKS, 60)	*S. lividans* TK21	• ∼200 kb gDNA fragments could be inserted into shuttle vector with high efficiency	• Challenging to achieve unique restriction sites	(Liu et al., 2009; Nah et al., 2015; Luo et al., 2016; Pyeon et al., 2017)
			*S. coelicolor* M145			
	• Homologous recombination and ligation	Tautomycetin (PKS, 80)	*S. coelicolor* M145		• Fragments must be free of such restriction sites	
			*S. lividans* TK21			
		Meridamycin (PKS, 95)	*S. lividans* TK24/K4-114			
LCHR, LLHR	• Recαβ-mediated linear to circular homologous recombination	Gougerotin (other, 17.6)	*S. coelicolor* M1146	• Rapid (2–3 days) and do not rely on PCR amplification	• Difficult to clone large size of DNA fragments	(Jiang et al., 2013; Yin et al., 2015; Yin et al., 2016; Qian et al., 2020)
	• RecET-mediated linear to linear homologous recombination	Streptoketides (PKS, 21.7)	*S. coelicolor* M1152/M1154			
		Oxytetracycline (PK, 29)	*S. venezuelae* WVR2006	• Suitable for small size of DNA fragments		
ExoCET	• *In vivo* RecET mediated homologous recombination with *in vitro* activities of T4 polymerase	Salinomycin (PKS,106)	*S. coelicolor* A3 (2)	• Application of a wide range of fragment size and genome complexities	• Efficiency is not so high	Wang et al. (2018)
TAR	• *In vivo* homologous recombination of *S. cerevisiae*	Enterocin (PK, 21)	*S. lividans* TK23	• Suitable for cloning various size of DNA fragments	• Some clones are unstable in yeast, so undergo deletions during mitotic propagation in yeast cells	(Yamanaka et al., 2014; Bonet et al., 2015; Tang et al., 2015; Bilyk et al., 2016; Novakova et al., 2018)
			*S. coelicolor* M1146			
		Grecocycline (PKS, 36)	*S. albus* J1074			
		Mithramycin A (PK, 45)	*S. lividans* TK24	• Highly efficient recombination system		
		Taromycin A (NRPS, 67)	*S. coelicolor* M145			
DNA assembler	• PCR-amplified small DNA fragments and vectors are co-transformed into *S. cerevisiae* and *in vivo* assembled	PTMs (PKS/NRPS, 18)	*S. lividans* 66	• Refactoring for cryptic gene clusters in a single-step manner with high efficiency	• Difficult to clone large size of DNA fragment	(Luo et al., 2013; Shao et al., 2013; Shao and Zhao, 2013)
		Aureothin (PK, 29)	*S. lividans*			
		Spectinabilin (PK, 45)	*S. lividans* 66			
Gibson assembly	• Two adjacent DNA fragments with same terminal sequences overlap to become one fragment by 5′ exonuclease, DNA polymerase and DNA ligase	Bicyclomycin (other, 9)	*S. coelicolor* M1146/M1152	• Assembled DNA molecules up to 100 kb in *E. coli* with low error rates	• Inefficiency for large size of DNA fragments	(Zhou et al., 2015a; Linares-Otoya et al., 2017; Vior et al., 2018)
		Kocurin (RiPP, 12)	*S. coelicolor* M1146	• Not rely on restriction enzyme site		
		Conglobatin (NRP, 41)	*S. coelicolor* M1154			
DiPAC	• Long amplicon PCR and *in vitro* HiFi assembly with Q5 polymerase	Hapalosin (PK/NRP, 23)	*E. coli* BAP1	• Simultaneous cloning and refactoring of BGCs are possible	• Time-consuming for cloning and costly cloning reagents	(D’agostino and Gulder, 2018; Greunke et al., 2018)
		Anabaenopeptin NZ857 (NRP, 29)	*E. coli* BAP1			
		Erythromycin (PK, 54.6)	*S. coelicolor* M1152/M1154			
CATCH	• RNA-guided Cas9 nuclease excision and Gibson assembly	Septacidin (other, 24)	*S. albus* J1074	• Various size of any DNA fragments can be extracted and assembled in a single step	• Efficiency is not so high	(Tang et al., 2018 Tao et al., 2019a)
		Tü3010 (PK/NRP, 27.4)	*S. avermitilis* MA-4680			

Plasmid *Streptomyces* bacterial artificial chromosome (pSBAC) is an *E. coli*-*Streptomyces* shuttle vector that can carry large BGCs. After insertion of the pSBAC vector into the restriction sites at the flanking regions of the BGC, the BGC could be separated from the genome by the restriction enzymes and captured into pSBAC (Nah et al., 2015). This vector can switch conveniently from single-copy to high-copy replication in *E. coli* and then integrate into heterologous *Streptomyces* host after intergenic conjugation (Liu et al., 2009). When integrated, pSBAC used phage ΦBT1 *attP*-*int* site-specific integration system instead of a ΦC31 *attP*-*int* system, which has been reported to have detrimental effects on antibiotic production. Using this method, meridamycin (Liu et al., 2009), tautomycetin (Nah et al., 2015), and pikromycin (Pyeon et al., 2017), which are large BGCs, were successfully expressed. However, during the process, target BGCs must be free of restriction sites, and sometimes it is challenging to find unique restriction sites.

Both linear-to-circular homologous recombination (LCHR) and linear-to-linear HR (LLHR) are strategies in which homologous recombination occurs between the flanking sequences of the target BGC from digested genomic DNA and the vector containing homology arms after co-transformation into *E. coli* (Ma and Wang, 2019). LCHR occurs between the linear insert and the circular vector using the Red-α/β protein derived from the lambda red phage, whereas LLHR occurs between two linearized DNA fragments using RecE/T from a Rac prophage (Fu et al., 2012). These methods are rapid and highly efficient for cloning small- or medium-sized BGCs, such as gougerotin (Jiang et al., 2013), streptoketides (Qian et al., 2020), and oxytetracycline (Yin et al., 2016). However, this is limited because the linearized vector and BGC segment must be introduced simultaneously into *E. coli* before homologous recombination occurs. Recently, exonuclease combined with RecET recombination (ExoCET), an improved strategy from LLHR, was developed (Wang et al., 2018). Because of the activity of 3′ exonuclease from *in vitro* T4 polymerase, approximately 80 bp single-strand homologous arms were generated from the flanking regions of a linear vector and a target BGC fragment. Two DNA fragments were annealed *in vitro* before transformation into *E. coli*. Owing to the *in vitro* annealing step, the efficiency of *in vivo* homologous recombination through the RecE/T system could be improved. Using ExoCET, 106 kb salinomycin was cloned without further assembly processes (Yin et al., 2015; Wang et al., 2018).

Unlike the above methods, transformation-associated recombination (TAR) cloning is a method in which the insert and vector are co-transformed into yeast. Yeast is known to have excellent recombination machinery, so it can clone large DNA fragments of up to 250 kb in size (Kouprina and Larionov, 2006). After cloning the linearized shuttle vector pCAP01 and the DNA fragment through homologous recombination in yeast, the vector was transformed into *E. coli*, transformed with *Streptomyces* by conjugation, and inserted into the chromosome via the *attP*-*int* site. However, some isolated clones may be unstable in yeast or lost during the mitotic propagation of yeast, while some DNA fragments may not be fully recovered in yeast (Kouprina and Larionov, 2006).

The DNA assembler is another *in vivo* assembly method in yeast based on TAR cloning (Shao et al., 2012). All successive DNA fragments were designed to overlap with each other and amplified by PCR. When fragments and a linearized vector were co-transformed into yeast, they were assembled into a vector with *in vivo* homologous recombination (Shao et al., 2009). This strategy has the advantage of modularity, which makes it possible to refactor gene clusters. In particular, by refactoring the well-known promoter, RBS, and terminator regions with the gene cluster using overlap extension PCR, silent BGCs could be expressed (Shao et al., 2013; Joska et al., 2014).

In addition to *in vivo* cloning, BGCs can also be cloned *in vitro*. *In vitro* cloning involves DNA assembly, which is mainly based on Gibson Assembly. Gibson Assembly assembles several fragments into one fragment by overlapping adjacent DNA fragments with the same terminal sequences using a 5′ exonuclease, DNA polymerase, and ligase (Gibson et al., 2009). Using this method, short-and medium-sized BGCs were efficiently cloned (Zhou et al., 2015a; Linares-Otoya et al., 2017). Recently, to compensate for the decrease in efficiency due to high GC content, a modified Gibson Assembly was developed, and 67 kb of BGC was successfully cloned (Li et al., 2015; Li et al., 2018).

Direct pathway cloning (DiPaC) is an *in vitro* cloning method based on Gibson Assembly. After making a long amplicon PCR of approximately 10 kb per each using Q5 high-fidelity polymerase, the fragments were assembled into a complete BGC using HiFi DNA assembly and captured as a pET28 vector (Greunke et al., 2018). This strategy is suitable for small- or medium-sized BGCs; however, this is a time-consuming process and the reagent is expensive. Recently, a strategy has been introduced to overcome the limitations of DiPaC with sequence- and ligation-independent cloning (SLIC), using T4 polymerase instead of HiFi DNA assembly (D’agostino and Gulder, 2018).

The Cas9-assisted targeting of chromosome segments (CATCH) method is also an *in vitro* cloning method based on Gibson Assembly. All or partial fragments of a BGC are directly cleaved using PCR-based amplified single-guide RNA (sgRNA) and Cas9 protein and then captured into a vector using Gibson Assembly, the whole process being performed at once on agarose gels (Jiang and Zhu, 2016). Since only the target region is cleaved using the Cas9 protein, intact fragments without an off-target could be captured into vectors. In addition, the risk of DNA shearing is low compared to other methods because chromosomes are protected by the agarose matrix. Thus, even very large fragments of approximately 100 kb can be cloned (Jiang et al., 2015).

*In vivo* cloning strategies can clone large fragments with high efficiency, but since cloning has to be done in bacteria or yeast, the process is slow because it has to wait for cells to grow. Furthermore, extraction from organisms is necessary to achieve the vector, which is then transformed into *E. coli* to transfer them to *Streptomyces* by conjugation. On the other hand, since *in vitro* cloning is not performed in cells, the process proceeds easily and quickly. However, the efficiency of cloning large DNA fragments is not yet high. If *in vivo* and *in vitro* strategies can be used together to compensate for their respective disadvantages, large BGCs can be cloned more efficiently and maintained in stable conditions.

### Genetic Parts

The development of efficient cloning tools has made it possible to introduce target BGCs into heterologous hosts. However, after being introduced into a heterologous host, only trace amounts of target SMs were detected. This is because the expression of BGCs is regulated by complex regulatory networks (Xia et al., 2020). Therefore, attempts have been made to engineer additional genetic parts to express target BGCs (Table 2).

**TABLE 2:** Examples of genetic parts for gene expression.

Vector	Strategies	Name	Reference
*Replicative*	Low-copy	pRM5, pHU204, pOJ446, pIJ101	(Marti et al., 2000; Medema et al., 2011; Carrillo Rincon et al., 2018)
	High-copy	pUC119, pKC1139	Du et al. (2015)
*Integrative*	ΦC31 *attP-int* locus	pSET152, pOJ436, pIJ6902, pIJ10702 (cosmid), pHL931 (BAC), pESAC13 (BAC), pCAP01 (TAR cloning)	(Huang et al., 2005; Jones et al., 2013; Bonet et al., 2015; Yin et al., 2016; Horbal et al., 2018a; Liu et al., 2018b)
	ΦBT1 *attP-int* locus	pMS82, pJ10257, pSBAC	(Gregory et al., 2003; Hong et al., 2005; Liu et al., 2009)
	VWB *attP-int* locus	pCLY10 (TAR cloning)	Bilyk et al. (2016)
**Genetic parts**		**Features**	**Reference**
Integrase	ΦC31	Derived from *Streptomyces* phage ϕC31	Khaleel et al. (2011)
	ΦBT1	Derived from *Streptomyces* phage ϕBT1	(Baltz, 2012 Zhang et al., 2013)
	VWB	Derived from bacteriophage VWB, introduced to a tRNA^Arg^ (AGG) gene in several *Streptomyces* species	Van Mellaert et al. (1998)
	RP3	Derived from *Streptomyces* phage RP3, introduced to a tRNA^Arg^ (AGG) gene in *S. rimosus*	Van Mellaert et al. (1998)
	R4	Derived from *S. parvulus* phage R4, site-specifically introduced into the chromosome of *S. parvulus*	Foor et al. (1985)
	TG1	Derived from *Streptomyces* phage TG1, site-specifically introduced in *S. cattleya*	Foor et al. (1985)
	Bxb1	Derived from Mycobacteriophage Bxb1	Ghosh et al. (2006)
	SV1	Derived from *S. venezuelae* phage SV1	Fayed et al. (2014)
	Φ1/6	Derived from prophage Φμ1/6, introduced to chromosome of tetracycline producing strains, *S. aureofaciens*	Farkasovska and Godany, (2012)
	ΦOZJ	Derived from actinophage OzzyJ	Ko et al. (2020)
	ΦJoe	*Streptomyces* phage ϕJoe, introduced to SCO2603, an ancestral phage fragment, in *S. coelicolor*	Fogg et al. (2017)
Constitutive Promoter	*ermEp*	Derived from *S. erythraeus,* promoter of erythromycin resistance gene	Alexander et al. (2010)
	ermEp*	Trinucleotide deletion in the *ermEp1* region of *ermEp*	Alexander et al. (2010)
	SF14	Derived from phage 119 isolated from *S. ghanaensis*	Labes et al. (1997)
	*gapdhp*	Derived from *S. griseus*, promoter of glyceraldehyde-3-phosphate dehydrogenase	Shao et al. (2013)
	*rpsLp*	Derived from *S. griseus,* promoter of 30S ribosomal protein	Tan et al. (2017)
	*kasOp*	Derived from *S. coelicolor* A3, promoter of SARP family regulator	Wang et al. (2013)
	kasOp*	Engineered by removing the binding sites of ScbR and ScbR2 regulators, then by using random mutation	Wang et al. (2013)
	*thlM4p*	Derived from *S. chattanoogensis* L10, promoter of peptidase M4 thermolysin	Wang et al. (2019a)
	Synthetic promoter library	Randomized to construct synthetic promoter library in *S. lividans,* 38 synthetic promoters sequenced	Seghezzi et al. (2011)
		Randomized synthetic promoter library in *S. venezuelae.* Among 180, six showed stronger strength than kasOp*	Bai et al. (2015)
		Synthetic promoter library based on *ermEp1* promoter. Among 56, one promoter shows stronger strength than *ermEp1*	Siegl et al. (2013)
Inducible promoter	*tipAp*	Thiostrepton inducible promoter from *S. lividans* 66	Chiu et al. (1999)
	*tcp830p*	Tetracycline inducible promoter	Rodriguez-Garcia et al. (2005)
	*nitAp*	Isovaleronitrile or ε-caprolactam inducible promoter from *Rhodococcus rhodochrous* J1	Komeda et al. (1996)
	*xylAp*	Xylose inducible promoter	Noguchi et al. (2018)
	*otrBp*	Oxytetracycline inducible promoter	Yang et al. (2019)
	PA3*-rolO-*RolR	Resorcinol inducible promoter	Horbal et al. (2014)
	P21-*cmt*-CymR	Cumate inducible promoter	Horbal et al. (2014)
Terminator	TD1	Derived from *Bacillus subtilis* phage Φ29	Pulido et al. (1987)
	Fd	Derived from *E. coli* phage fd	Ward et al. (1986)
	*ttsbiB*	Heterologous expression level of glucuronidase in Mycobacteria	Horbal et al. (2018b)
Riboswitch		Synthetic theophylline-dependent riboswitches	Eyles et al. (2018)

Vectors can be divided into two types depending on whether they self-replicate in the host cell or integrate into the host genome. First, replicative vectors are mainly derivatives based on the pRM5 vector containing the SCP2 replicon (Marti et al., 2000). This backbone vector is maintained in *Streptomyces* in a low-copy state and can be stably replicated up to a size of 31 kb or more (Fong et al., 2007). Meanwhile, pKC1139 is mainly used to improve the productivity of target BGCs while maintaining 20–50 copy numbers in *Streptomyces* (Du et al., 2015). Since BGCs are large in size, low-copy replicative vectors were used to maintain stability within *Streptomyces* (Marti et al., 2000). However, as these vectors also have some limitations, such as difficulties in genetic manipulation and reduced DNA purity, due to a low level of DNA recovery, the strategy was changed from a replicative vector to an integrative vector (Liu et al., 2009; Heidorn et al., 2011). ΦC31 integrase is commonly used, but the problems of detrimental effects on the production of BGCs and entering a pseudo position have been reported in some strains. Therefore, ΦBT1 replaced ΦC31. In addition, several other integrases were identified (Table 2). Recently, a modular and integrative vector that is easily compatible with vectors for cloning and assembly methods has been developed (Aubry et al., 2019). The antibiotic resistance cassette module and the integration system cassette module can be easily replaced with other cassettes by unique restriction sites, so multiple vector types can be generated from one backbone. Because it has the advantage of being able to add a module, this method can contribute to resolving the difference in efficiency depending on the type of host or cloning strategy (Aubry et al., 2019).

One of the most critical factors for expressing target BGCs is the promoter element. First, constitutive promoters are commonly used for overexpression of heterologous genes. In particular, the *ermE* promoter, which contains two regions, *ermEp1* and *ermEp2*, is widely used as a strong constitutive promoter. ermEp* is a variant that is upregulated by the deletion of trinucleotides from the *ermEp1* region (Alexander et al., 2010). In addition to the *ermE* series, there are several strong constitutive promoters, SF14, *rpsLp, gapdhp*, and *kasOp*, each of which is known to have similar or stronger intensities to ermEp* (Labes et al., 1997; Shao et al., 2013; Wang et al., 2013). *kasOp* was engineered by removing two binding sites of regulators and then using random mutation. Finally, kasOp* was developed, which showed the strongest strength in *S. coelicolor* and *S. avermitilis* compared to SF14 and ermEp*, because its structure can be recognized more readily by sigma factor, HrdB, than the other two promoters (Wang et al., 2013).

Promoters have different strengths depending on the species. To overcome this limitation, a method was developed to screen strong constitutive promoters among synthetic promoter libraries by randomizing the consensus sequences of known promoters (Seghezzi et al., 2011; Siegl et al., 2013; Bai et al., 2015). For example, to acquire stronger promoters than kasOp*, a library was constructed by mutating the spacer sequence between the -35 and -10 regions of kasOp* in *S. venezuelae*, and their strengths were measured by GFP fluorescence. Among the 180 synthetic promoters, six synthetic promoters were stronger than kasOp* (Bai et al., 2015). Alternatively, an approach to discover strong native promoters within the host has been developed. For example, to expand a panel of strong native promoters, 32 candidates that may be strong promoters were screened using RNA-seq data from *S. albus*. Then, through *xylE* activity assay with time course analysis, 10 native promoters were validated to have stronger strength than kasOp* (Luo et al., 2015).

The disadvantage of constitutive promoters is that sustained expression of BGCs can generate negative effects on host bacteria, such as products becoming toxic or unable to maintain the bacterial growth rate. Inducible promoters have the advantage of being able to turn on gene expression only at a desired point. However, some inducible promoters have the disadvantage of leaky expression (Huang et al., 2015), such as the commonly used *tipA* promoter (Chiu et al., 1999) induced by thiostrepton and *tcp830* strong promoter (Rodriguez-Garcia et al., 2005) induced by tetracycline. To overcome the problem of leaky expression of promoters, synthetic inducible systems have been developed. One is the PA3-*rolO*-RolR system, which consists of a codon-optimized RolR regulator and synthetic promoter A3 with operater *rolO* from *C. glutamicum*, induced by resorcinol. The other is the P21-*cmt*-CymR system, similar to the resorcinol inducible system, induced by the presence of cumate, by binding CymR to the *cmt* operator fusing with the synthetic promoter 21 (Horbal et al., 2014).

The transcriptional terminator is also an essential genetic component for gene expression to prevent readthrough problems. Terminators mainly consist of inverted repeat sequences, which are known to contribute to stability by forming a stem-loop structure in the mRNA state. However, only a limited number of terminators are efficient in *Streptomyces*. The TD1 terminator, derived from *B. subtilis* phage Φ29, successfully recognized the termination of *S. lividans* and was active as an *in vivo* terminator (Pulido et al., 1987). Likewise, the Fd terminator originating from *E. coli* phage fd can also be used as an *in vivo* terminator (Ward et al., 1986). Another example is the synthetic bi-directional transcriptional terminator B (*ttsbiB*) is a highly efficient terminator in *S. lividans* in the test of heterologous glucuronidase activity (Huff et al., 2010; Horbal et al., 2018b).

The riboswitch is composed of an aptamer region that detects a compound and a gene expression control region that can change the structure after the aptamer binds. By redesigning sequences that interact with aptamers, a synthetic riboswitch was developed that can initiate the transcription when a specific compound enters (Rudolph et al., 2013). Based on this principle, a theophylline-dependent riboswitch introduced in *S. coelicolor* increased heterologous bottromycin production by 120-fold (Eyles et al., 2018). In addition, efforts have been made to increase or control the efficiency of gene expression by using genetic parts for translational regulation, such as RBS and codon usage (Makrides, 1996). Nevertheless, since many genetic parts still show different efficiencies depending on *Streptomyces* species, continuous development of genetic parts is required.

## Chassis Development for Rational Engineering BGC Heterologous Expression in *Streptomyces*

The expression of BGCs in native hosts might be difficult in genetic engineering, have a complex metabolic background, and their endogenous BGCs are often cryptic. Then, the producers showed no expression of BGCs under laboratory conditions. Therefore, BGC expression using a heterologous production platform is a solution. The key characteristics for robust heterologous expression chassis are as follows: 1) fast growth, 2) genetic amenability and well-established genetic toolkits, 3) clean metabolic background to supply precursors for building a variety of SMs and express diverse biosynthetic classes with minimal interruption from host SMs; 4) providing all of the genes of heterologous biosynthetic pathways regarding transcription, translation, and post-translational modifications, 5) resistance to SMs, effective efflux pump system, 6) efficient nutrient and oxygen utilization, and 7) optimization of downstream processes. Based on these principles, many chassis strains have been constructed in diverse species (Table 3).

**TABLE 3:** Examples of *Streptomyces* heterologous expression chassis.

Chassis	Genetic manipulation	Natural product	Product type	Effect	Reference
*S. coelicolor* M1146	• Deletion of four endogenous BGCs (Act, Red, Cpk, and CDA) from the genome of *S. coelicolor* M145, a derivative of *S. coelicolor* A3 (2) strain lacking two plasmids	Chloramphenicol, congocidine, cypemycin, grisemycin, actagardine, planosporicin, GE37468, napsamycin, clorobiocin, coumermycin A1, caprazamycin, FK506/FK520 (tacrolimus), merochlorins, gougerotin, endophenazine, roseoflavin, holomycin, and tunicamycin	NRP, PK, linaridin, RiPP, oligopyrrole, aminocoumarin, and other	• Reduced competition for precursor	(Gomez-Escribano and Bibb, 2011; Gomez-Escribano and Bibb, 2014; Bekiesch et al., 2016)
				• High conjugation frequency	
*S. coelicolor* M1152	• Introduction of point mutations in *rpoB* [S433L] in *S. coelicolor* M1146			• Higher transcriptional and translational fidelity	
				• Clean metabolic background	
*S. coelicolor* M1154	• Introduction of point mutations in *rpsL* [K88E] in *S. coelicolor* M1152			• Induction of global upregulation of SM biosynthesis	
				• Production of chloramphenicol and congocidine 40-, and 30-fold than *S. coelicolor* M145, respectively	
*S. coelicolor* M1317	• Deletion of all three Type III PKS genes (*gcs*, *srsA*, *rppA*) and operons from *S. coelicolor* M1152	Flaviolin	PK	• Specialized expression host for actinobacterial type III PKS genes	Thanapipatsiri et al. (2015)
*S. coelicolor* ZM12	• Deletion of all the 10 PKS and NRPS BGCs and a 900 kb subtelomeric sequences from the genome of *S. coelicolor* M145	Galbonolide B	PK	• Reduced competition for precursor	(Zhou et al., 2012; Liu et al., 2015)
*S. lividans* SBT5	• Deletion of Act, Red, and CDA BGCs from *S. lividans* TK24	Murayaquinone, hybrubins, and Whole genome BAC library from *S. rochei*	PK and PKS-NRPS hybrid	• Positive regulation of *afsRS* gene on cryptic BGC genes expression	Xu et al. (2016)
	• Introduction of the global regulatory genes (*afsRS*_*cla*_) from *S. clavuligerus*				
*S. lividans* GX28	• Stepwise integration of two global regulatory genes (*nusG*_*SC*_, *afsRScla*) and two codon-optimized multi-drug efflux pump genes (*lmrA*, *mdfA*) into *S. lividans* SBT5	Murayaquinone, hybrubins, dehydrorabelomycin, piericidin A1, and actinomycin D	PK, PKS-NRPS hybrid, and NRP	• Superior host for high-throughput heterologous expression of BGCs and LEXAS screening	Peng et al. (2018)
				• 74 times higher yields of murayaquinone than that of SBT5	
*S. lividans* LJ1018	• Deletion of negative regulatory gene (*wblA*_*sl*_) from *S. lividans* SBT5			• Increased heterologous production of PKs, NRPs, and hybrid antibiotics	
	• Introduction of global regulatory gene (*afsRS*_*cla*_) and two codon-optimized multi-drug efflux pump genes (*lmrA*, *mdfA*)			• Positive morphological change	
				• The yields of murayaquinone were 96 times higher than that of SBT5 (10.6 mg/L)	
*S. lividans* ΔYA9	• Deletion of 9 endogenous BGCs (178.5 kb) within the chromosome of *S. lividans* TK24 using iterative marker excision system (IMES)	*S. albus subsp. chlorinus* NRRL B-24108 genomic library	Library	• Increased success rate in isolation of novel bioactive NPs originating from eDNA	Ahmed et al. (2020)
	• Introduction of two additional phage phiC31 *attB* loci				
*S. lividans* ΔYA11	• Deletion of 11 endogenous BGCs (228.5 kb)	Tunicamycins, deoxycoformycin, deoxycinnamycin, and 7 new compounds	Nucleoside antibiotic, lanthipeptide, and aromatic polyketide	• Used in the production of amino acid-based natural products	
	• Introduction of two additional phage phiC31 *attB* loci				
*S. albus* Del14	• Deletion of 15 endogenous BGCs using IMES from *S. albus* J1074, defective in an active SalGI restriction-modification system (500 kb) (Cluster-free J1074)	Cinnamycin, tunicamycin, didesmethylmensacarcin, fralnimycin, bhimamycin A, aloesaponarin II, albucidin, cittilinsn, and *S. albus* subsp. chlorinus NRRL B-24108 genomic library	PK, RiPP, and new compound	• Clean metabolic background	Myronovskyi et al. (2018)
				• The activation of the cryptic BGCs from *Streptomyces*. sp. and *Frankia* sp	
*S. albus* B4	• Introduction of two additional phage phiC31 *attB* sites into the chromosome for stability (total four *attB* sites)	Tunicamycin B2, moenomycin M, didesmethyl mensacarcin, demethoxyaranciamycinone, griseorhodin, and cinnamycin	PK, saccharide, and lantipeptide peptide	• Reduced competition for precursor	
				• Multi-copy integration	
*S. albus* ZXJ-6	• Introduction of a three-gene cassette for the biosynthesis of ethylmalonyl-CoA and salinomycin	Actinorhodin	PK	• Host for heterologous production of PK	Zhang et al. (2017b)
	• Subsequent deletion of the salinomycin BGC			• Rich supplies of common PK precursors including malonyl-CoA, methylmalonyl-CoA, and ethylmalonyl-CoA	
				• Enhanced intracellular energy (ATP) and reducing power (NADPH/NADP+)	
*S. avermitilis* SUKA5	• Deletion of 1.51 Mb left arm (two majors endogenous BGCs) and oligomycin BGC from *S. avermitilis* WT (82.11%)	Pladienolide	PK	• Clean metabolic background	Komatsu et al. (2010)
				• Increase in genetic stability	
				• Reduced competition for precursor	
				• Large amount of cell mass	
				• Functionalization of positive regulator gene	
				• Capable of expressing diverse BGCs	
*S. avermitilis* SUKA17	• Deletion of three terpene compound BGCs (geosmin, neopentalenolactone, and carotenoid) from *S. avermitilis* SUKA5 (81.46%)	Cephamycin, and amorpha-1,11-diene	NRP and plant terpenoid intermediate	• Acyl-CoA precursor pool supply	
				• Production of unnatural metabolites by combinatorial biosynthesis	
				• PKS production at the industrial level	
*S. avermitilis* SUKA22	• Isogenic to SUKA17, the right side of the deletion region of SUKA17 was replaced by a mutant-type *loxP* sequence to prevent undesired recombination	Shinorine, porphyra-334, 17-hydroxycyslabdan A, raimonol, pholipomycin, resistomycin, bafilomycin B1, and nemadectin	NRP, PK, terpene, and other	• Positive morphological change	(Komatsu et al., 2013; Bekiesch et al., 2016)
*S. chattanoogensis* L321	• Deletion of 0.7 Mb non-essential genomic region (7 putative BGCs and complete natamycin BGC)	eGFP, indigoidine, and actinorhodin	NRP and PK	• Enhanced ATP and reducing power	Bu et al. (2019)
				• Improved productivity of protein and secondary metabolite	
				• Positive morphological change	
				• Clean metabolic background	
				• Increase in genetic stability	
				• Promising platform cell to produce PK	
*S. venezuelae* YJ003	• Deletion of all *des* gene clusters (*desI*-*desVIII* and *desR*) from the wild type	Gentamicin A2, kanamycin	Aminoglycoside antibiotics	• Used in the production of polyketides and aminoglycosides	Hong et al. (2004)
*S. venezuelae* YJ028	• Deletion of both *pikA* PKS genes and *des* genes	Doxorubicin	PK	• Used in the sugar engineering	Jung et al. (2007)
*S. venezuelae* DHS2001	• Deletion of *pikA* gene from wild type	Tylosin polyketide synthase, epothilones, flavonoid, stilbene, flavones, flavonols, barbamide, naringenin, pinocembrin, and 4-O-demethylbarbamide	PKS, plant-specific PK and lipopeptide (hybrid NRPS-PKS)	• Diverse precursors for PKs	(Jung et al., 2006; Park et al., 2009; Park et al., 2010; Park et al., 2011; Kim et al., 2015)
				• Used in the production of PKS, NRPS, and PKS-NRPS hybrid	
*S. venezuelae* WVR2006	• Deletion of jadomycin biosynthetic gene cluster	Oxytetracycline	PK	• Normal growth, differentiation	(Fan et al., 2012; Yin et al., 2016)
	• Downstream of Pik PKS (*pikAV*, *pikC*, *pikD*) remains intact			• Cleaner metabolite profiles	
				• Improved oxytetracycline production up to 430 mg/L in 48 h	
*S. rimosus* SR0	• Deletion of whole oxytetracycline gene cluster of *S. rimosu*s 461	Chlortetracycline	PK	• Several grams level of titer with industrial grade, one of the highest titer reports of heterologous antibiotics production	Wang et al. (2019b)
	• Introduction of constitutively expressed cluster-situated activator gene *ctcB*				

### S. coelicolor

*S. coelicolor* is a genetically well-characterized *Streptomyces* species. The 8.6 Mb genome has been reported, including over 20 BGCs (Bentley et al., 2002). *S. coelicolor* is the genetically best-studied species as the regulatory systems of endogenous BGCs has been well-understood. Genetic tools, including replicative and integrative vectors and well-established genetic parts, can be applied in *S. coelicolor*. Several mutant strains have been developed for successful heterologous expression of BGCs for the production of SMs. In addition to the deletion of the main antibiotic gene clusters (*S. coelicolor* M1146), point mutations in *rpoB* encoding RNA polymerase *β*-subunit (*S. coelicolor* M1152), and *rpsL* encoding ribosomal protein S12 were introduced to regulate the strain at both the transcriptional and translational levels (*S. coelicolor* M1154) (Hu et al., 2002; Ochi, 2007; Gomez-Escribano and Bibb, 2011). As a result, transcriptional and translational fidelity increased BGC expression, and SM productivity was enhanced without growth impairment. These strains successfully expressed 18 different heterologous BGCs, leading to increased SM yields ranging from 0.4 to 160 mg/L. Moreover, the genome-minimized strain was constructed by deleting all the PKS and NRPS BGCs and also 900 kb sub-telomeric sequence (*S. coelicolor* ZM12) (Zhou et al., 2012). Galbonolide BGC was successfully expressed in heterologous *S. coelicolor* ZM12 with a clean background, which verified the essential role of core genes in the biosynthesis of galbonolides (Liu et al., 2015).

### S. lividans

*S. lividans* lacks an endonuclease restriction system, whereas *S. coelicolor* and *S. avermitilis* restrict methylated DNA, making it highly acceptable to foreign DNA. Notably, *S. lividans* presents high conjugation efficiency, and thus, this species is applicable for high-throughput transfer of the libraries (Martinez et al., 2004). Indeed, *S. lividans* TK24-derived strains have been used as heterologous hosts for library expression and function-directed screening systems (LEXAS) (Xu et al., 2016). *S. lividans* SBT5 was developed by the deletion of Act, Red, and CDA BGCs from *S. lividans* TK24 (Shima et al., 1996). For high-throughput heterologous expression and screening of genomic libraries to express cryptic BGCs and to mine bioactive compounds, SBT5 was further developed by elevating conjugation efficiency and the positive effects of global activators (Edgar and Bibi, 1997; Poelarends et al., 2002; Behnken et al., 2012; Peng et al., 2018). For example, *S. lividans* GX28 was used as a library expression host (Peng et al., 2018; Nah et al., 2021). As a result, high-throughput LEXAS of one BAC library and two cosmid libraries from three different *Streptomyces* strains successfully screened three antibiotic BGCs (Peng et al., 2018). In addition, a genome-minimized strain was constructed for cleaner and simpler metabolite profiles than the parental strain, in which 11 endogenous BGCs were deleted and two additional phage ФC31 *attB* sites were introduced (*S. lividans* ΔYA11) (Ahmed et al., 2020). The benefit of adding additional integration sites was validated by expressing heterologous gene clusters in both parental *S. lividans* TK24 and ΔYA11, whose production levels were elevated in *S. lividans* ΔYA11 by approximately two-fold.

### S. albus

*S. albus* is one of the most widely used heterologous expression hosts in *Streptomyces*. It provides successful heterologous expression of diverse BGCs for the production of PKs, NRPs, terpenes, and saccharides with high productivity (Wendt-Pienkowski et al., 2005; Gullon et al., 2006; Lombo et al., 2006; Winter et al., 2007; Feng et al., 2009; Makitrynskyy et al., 2010; Bilyk and Luzhetskyy, 2014). The versatility of *S. albus* is highly related to its relatively small genome (6.8 Mb) and the availability of efficient genetic toolkits (Zaburannyi et al., 2014)*. S. albus* J1074 is a derivative of the *S. albus* G1 defective in an active SalI restriction-modification system; thus, heterologous BGCs can be easily transformed. *S. albus* J1074 mostly showed the best performance in isomigrastatin (PK) production, which was 2 to 10-fold higher than that of other *Streptomyces* chassis strains (Yang et al., 2011). The remarkable production capacity was also demonstrated by discovering novel SMs from cryptic gene clusters of the metagenome, which did not yield on other chassis hosts. From *S. albus* J1074, all dispensable BGCs, including PKS, NRPS, lanthipeptide, and glycoside antibiotic clusters, were gradually deleted and marker-free; thus, an extremely clean metabolite profile was achieved, named *S. albus* Del 14. Additionally, ФC31 *attB* sites were introduced for multi-copy integration (*S. albus* B4). This large deletion did not influence growth, morphological characteristics, or fitness.

### S. avermitilis

*S. avermitilis* is an industrial microorganism that produces important anthelmintic agent avermectins (Miller et al., 1979; Gao et al., 2010). *S. avermitilis* SUKA5, SUKA17, and SUKA22 strains were genome-reduced derivatives from the wild-type. *S. avermitilis* has an intrinsically stable genome because it has the shortest terminal inverted repeats (TIR) (Komatsu et al., 2013). Such genetic properties are strengthened by systematic large-scale deletions; thus, the strain is more suitable for the expression of exogenous BGCs (Komatsu et al., 2010; Komatsu et al., 2013). The difference in pladienolide production between wild-type and SUKA5 is more than 20-fold, which appears to be due to the competition for the common acyl-CoA precursor for pladienolide biosynthesis and avermectin biosynthesis in the wild-type strain, which demonstrates the extended precursor availability in mutants. In addition, morphological differentiation, growth rate, and biomass in the stationary phase were enhanced compared to the wild-type strain (Komatsu et al., 2013). The engineered host can produce heterologous PKs, NRPs, aminoglycosides, shikimate-derived compounds, and terpenes.

### Others

*S. chattanoogensis* is an industrial microorganism used for the production of natamycin. Through the rational deletion of non-essential genomic regions based on systematic analysis, a genome-reduced chassis strain was constructed (*S. chattanoogensis* L321) (Zhou et al., 2015b). Unlike wild-type, the engineered strain does not have an endogenous CRISPR/Cas system; therefore, several CRISPR/Cas9 systems were successfully introduced without any interference and improved the efficiency of genome editing. Heterologous production level of SMs in wild-type and the chassis was investigated during serial passages of the culture, resulting in the constant level in the chassis, while the reduced level in the wild-type. This is because the undesired mutations were generated and accumulated in the wild-type genome according to the serial passages, while the removal of mobile genetic element in the chassis genome showed a positive effect on genetic stability. Also, the rational deletion of non-essential genomic regions might pleiotropically influence the engineered strain, resulting in highly efficient expression of BGCs. Indeed, the *S. chattanoogensis* chassis strain showed great performance as a heterologous host, especially in PKs (Zhou et al., 2015b).

*S. venezuelae* ATCC 15439 has a fast growth rate, which enables a large accumulation of cell mass and metabolites (Xue and Sherman, 2001). In addition, ease of genetic manipulation, liquid sporulation in a dispersed manner, and abundant building blocks for SMs are other advantages. Three major chassis strains of *S. venezuelae* are 15,439, DHS 2001, YJ003, and YJ28. These chassis strains are promising heterologous hosts that successfully express diverse heterologous BGCs from different sources.

*S. rimosus* 461 is a high-yielding industrial producer of oxytetracycline. Therefore, the construction of a heterologous host from *S. rimosus* 461 is worthwhile for expression of BGCs for the production of other tetracycline antibiotics and type II PK, validated by the heterologous expression of chlortetracycline BGC in SR0 chassis, which is 38-fold higher than that of the original producer, and 68-fold higher than that of the *S. lividans* strain (Wang et al., 2019b).

Furthermore, a rational study using computational approaches, such as comparative genome analysis, should be widely used for the prediction of dispensable genetic elements, such as mobile genetic elements, genomic islands, and BGCs, to carefully engineer the strains for heterologous expression of BGCs. In addition to BGC expression, elimination of the competing precursor sinks greatly facilitates the identification of exogenous bioactive compounds, improves production yield with increased precursor pool, and streamlines downstream processing.

## Rational Engineering Approaches for Heterologous Production of Recombinant Proteins in *Streptomyces*

As a decomposer in natural habitats, *Streptomyces* secretes multiple enzymes to degrade saprophytic compounds, and its secretion capacity makes *Streptomyces* attractive as a host for recombinant protein production (Crawford, 1978). In this section, the secretion pathways of *Streptomyces* and approaches to increase recombinant protein yield are discussed.

### Secretion Pathway of *Streptomyces*

The Sec-pathway is the dominant bacterial protein export pathway, comprising approximately 96% of exported proteins in *E. coli* (Orfanoudaki and Economou, 2014)*.* On the other hand, for *Streptomyces*, especially *S. lividans*, twin-arginine translocation (Tat-pathway) is exploited for approximately 21% of the secreted proteins (Tsolis et al., 2018). Utilization of the Tat-pathway, which secretes proteins in the folded state, can be advantageous over the Sec-pathway, since cytoplasmic folding is crucial for the activity of some proteins (Weiner et al., 1998; Feilmeier et al., 2000; Thomas et al., 2001). However, the Sec-pathway is generally superior to the Tat-pathway for heterologous protein production in terms of production titer and applicability. For example, the production yield of streptokinase from *Streptococcus equisimilis* in *S. lividans* was 30 times higher when the Sec-pathway was utilized (Kim et al., 2010). In addition, the overproduction of Sec-pathway dependent protein (α-amylase) using Tat-pathway was unsuccessful in *S. lividans*. (Gullon et al., 2015b). Since the Tat-pathway exports folded proteins, the secretion efficiency may be highly dependent on the structure of the protein (Fisher et al., 2008). To demonstrate the lower product yield *via* the Tat-pathway, a comparative transcriptomics approach was applied to *S. lividans* overexpressing proteins via either Sec-pathway or Tat-pathway to identify possible bottlenecks for protein production, and a stringent response was induced when the Tat-pathway was exploited for protein secretion (Gullon et al., 2015a). However, the Tat-pathway is still worth investigating for proper intracellular folding of proteins.

### Approaches to Increase the Protein Yield

*Streptomyces* would not be a preferred host over *E. coli* for heterologous protein production, with few exceptions, including proteins from Streptomycetes, due to the lower product yield (Kim et al., 2010). Many efforts have been made to improve protein production, and representative examples are presented in Table 4.

**TABLE 4:** Selected examples of optimization of heterologous protein production in *Streptomyces* since 2010.

Optimization	Product	Native host	Expression host	Plasmid	Promoter	Promoter host	Signal peptide	Signal peptide host	Host engineering	Reference
Gene expression	DagA (agarase)	*S. coelicolor* A3 (2)	*S. lividans* TK24	pWHM3-TR1R2	*sprT*p	*S. griseus*	Native			Temuujin et al. (2011)
				pHSEV-1	*tipA*p	*S. lividans*	Native			
				pUWL201 PW	*ermE*p	*Saccharopolyspora erythraea*	Native			
	SCO3487 (β-agarase)	*S. coelicolor* A3 (2)	*S. lividans* TK24	pUWL201 PW	*ermE*p	*Saccharopolyspora erythraea*	Native			Temuujin et al. (2012)
	PVA (penicillin V acylase)	*S. lavendulae* ATCC 13664	*S. lividans* 1,326	pEM4	ermE*p	*Saccharopolyspora erythraea*	Native			Torres-Bacete et al. (2015)
	BTA hydrolase	*Thermobifida* sp. BCC23166	*S. rimosus* R7	pIJ8600	*tipA*p	*S. lividans*	Native			Sinsereekul et al. (2010)
	Transglutaminase	*S. hygroscopicus* WSH03-13	*S. lividans* TK24	pIJ86	Native		Native			Liu et al. (2016)
					Native (negative regulatory element deletion)		Native		
							Native; codon-optimized		
	Lipase	Metagenomic	*S. lividans* 10-164	pIAFC109	C109p	-	Native			Cote and Shareck, (2010)
	Cel6A (endoglucanase)	*Thermobifida fusca* YX	*S. lividans* 1,326	pZRJ362	Xylose isomerase promoter	*Actinoplanes missouriensis*	Native			Li et al. (2013b)
	Phospholipase D	*S. halstedii* ATCC10897	*S. lividans* TK24	pIJ12739	*tipA*p/ermE*p dual promoter	*S. lividans*/*Saccharopolyspora erythraea*	Native			Tao et al. (2019b)
	Chitosanase	*Kitasatospora* sp. N106	*S. lividans* TK24	pHM8aBΔM	Native		Native			Dubeau et al. (2011)
					Native (negative regulatory element deletion)		Native			
					*S. ghanaensis* phage I19 derived promoter		Native			
					Native		Native		*csnR* deletion	
					Native (negative regulatory element deletion)		Native			
					*S. ghanaensis* phage I19 derived promoter		Native			
				pFDES	Native		Native			
					Native (negative regulatory element deletion)		Native		*csnR* deletion	
	Chitinase	Metagenomic	*S. lividans* TK24	pIJ86	ermE*p	*Saccharopolyspora erythraea*	Native			Berini et al. (2019)
			*S. venezuelae* ATCC10595							
			*S. coelicolor* A3 (2)							
	Glutenase	*Actinoallomurus* A8	*S. lividans* TK24	pIJ86	ermE*p	*Saccharopolyspora erythraea*	Native			Cavaletti et al. (2019)
	Chitobiase	*S. avermitilis* MA-4680	*S. lividans* 1,326	pIJ350	*xylA*p	*S. avermitilis* MA-4680	Native			Noguchi et al. (2018)
	Sfp2 (keratinase)	*S. fradiae* var. k11	*S. lividans* 1,326	pJTU4881	Xylose isomerase promtoer	*Actinoplanes missouriensis*	Native			Li et al. (2013a)
	O-glycoprotein	*Mycobacterium tuberculosis*	*S. lividans* 1,326	pIJ6021	*tipA*p	*S. lividans*	Native			Gamboa-Suasnavart et al. (2011)
Secretion system	DagA (agarase)	*S. coelicolor*	*S. lividans* TK21	pIJ486	Native		Native			Gabarró et al. (2017)
					Native		Native		*sipY* deletion	
	DagA (agarase)	*S. coelicolor*	*S. lividans* TK21	pAGAs1	Native		Native		WT	Gullon et al. (2015b)
									*secG* deletion	
									*tatC* deletion	
							*amlB*	*S. lividans* TK21	WT	
									*secG* deletion	
									*tatC* deletion	
Both	Transglutaminase	*S. mobaraensis*	*S. lividans*	pTONA4	*pld*p	*S. cinnamoneus*	*pld*	*S. cinnamoneus*	*-*	Noda et al. (2013)
	Pernisine	*Aeropyrum pernix* K1	*S. rimosus* M4018	pVF	*tcp830*p	Synthetic	*srT*	*S. rimosus*	*-*	Snajder et al. (2019)
							Native			
							Native; codon-optimized			
	Xys1 (xylanase)	*S. halstedii* JM8	*S. lividans* 1,326	pN702GEM3	Native		Native			Sevillano et al. (2016)
									*ssgA* overexpression	
							α-amylase	*S. griseus* IMRU 3570		
							α-amylase + 3 codons of the mature amylase			
					*pstS*p	*S. lividans*	Native			
					*xylA*p	*S. coelicolor*	Native			
					*glpQ*p	*S. coelicolor*	Native			
					ermE*p	*Saccharopolyspora erythraea*	Native			
					*vsi*p	*S. venezuelae* CBS762.70	Native			
	α-amylase	*S. griseus* IMRU 3570			*xysA*p	*S. halstedii*	Native			
									*ssgA* overexpression	
					*pstS*p	*S. lividans*	Native			
	Small laccase	*S. coelicolor*		pHJL401	xysA	*S. halstedii*	Native			
									*xlnR* deletion	
									*bxlR* deletion	
	XylE (catechol 2,3-dioxygenase)	*Pseudomonas putida*	*S. rimosus* M4018	pAB04	ermE*p	*Saccharopolyspora erythraea*	Native			Carrillo Rincon et al. (2018)
					*nitA*p	*Rhodococcus rhodochrous* J1	Native			
					*tcp830*p	Synthetic	Native			
				pVF	ermE*p	*Saccharopolyspora erythraea*	Native			
					*nitA*p	*Rhodococcus rhodochrous* J1	Native			
					*tcp830*p	Synthetic	Native			
				pVM	ermE*p	*Saccharopolyspora erythraea*	*vsi*	*S. venezuelae*	-	
							*amy*	*S. griseus*	-	
							*sprB*	*S. griseus*	-	
							*aml*	*S. venezuelae*	-	
							*xysA*	*S. halstedii*	-	
							aml*	*S. venezuelae*	-	
							*srT*	*S. rimosus*	-	
							*lip*	*S. rimosus*	-	
	AppA (phytase)	*Escherichia coli*		pVM	ermE*p	*Saccharopolyspora erythraea*	*srT*	*S. rimosus*	-	
							*lip*	*S. rimosus*	-	
							*aml*	*S. venezuelae*	-	
				pVF	*nitA*p	*Rhodococcus rhodochrous* J1	*srT*	*S. rimosus*	-	
					*tcp830*p	Synthetic			-	
				pAB04	ermE*p	*Saccharopolyspora erythraea*	*srT*	*S. rimosus*	-	
	CelA (cellulase A)	*Rhodothermus marinus*DSM4253	*S. lividans* TK24	pIJ486	*vsi*p	*S. venezuelae* CBS762.70	*vsi*	*S. venezuelae* CBS762.70	-	Hamed et al. (2017)
	hTNF-α	human	*S. lividans* TK24	pIJ486	*vsi*p	*S. venezuelae* CBS762.70	*vsi*	*S. venezuelae* CBS762.70	-	Lule et al. (2012)
									*pepck* overexpression	
	Interleukin-6	human	*S. lividans* TK24	pIMB1	ermE*p	*Saccharopolyspora erythraea*	*melC1*	*S. antibioticus*	-	Zhu et al. (2011)
							*cagA*	*S. globisporus* C-1027	-	
							*cagA* (TTA codon to CTG codon)	*S. globisporus* C-1027	-	
	Transglutaminase	*Streptoverticillium cinnamoneum*	*S. lividans* 1,326	pIJ702	*pld*p	*Streptoverticillium cinnamoneum*	*pld*	*Streptoverticillium cinnamoneum*	-	Noda et al. (2010)
	β-1,4-endoglucanase	*Thermobifida fusca* YX							-	
	β-glucosidase								-	
	Transglutaminase	*S. hygroscopicus* WSH03-13	*S. lividans* 1,326	pIJ86	Native		Native			Guan et al. (2015)
					Native		Native (TTA codon to CTG codon)			
					ermE*p	*Saccharopolyspora erythraea*	Native (TTA codon to CTG codon)			
			*S. griseus*		Native		Native (TTA codon to CTG codon)			
			*S. lividans* TK24		Native		Native (TTA codon to CTG codon)			
	Aminopeptidase	*Bacillus subtilis* Zj016	*S. lividans* TK24		Transglutaminase promoter	*S. hygroscopicus* WSH03-13	Transglutaminase (TTA codon to CTG codon)	*S. hygroscopicus* WSH03-13	-	
			*S. lividans* 1,326						-	
			*S. griseus*						-	
			*S. hygroscopicus* FR008						-	
	Phenylalanine ammonia-lyase	*Rhodotorula glutinis*	*S. lividans* TK24		Transglutaminase promoter	*S. hygroscopicus* WSH03-13	Transglutaminase (TTA codon to CTG codon)	*S. hygroscopicus* WSH03-13	-	
			*S. lividans* 1,326						-	
			*S. griseus*						-	
			*S. hygroscopicus* FR008						-	
	Streptokinase	*Streptococcus equisimilis*ATCC 9542	*S. lividans* TK24	pWHM3-TR1R2	*sprT*p	*S. griseus*	*sgt*	*S. griseus*	-	Kim et al. (2010)
							Native			
				pUWL201PW	ermE*p	*Saccharopolyspora erythraea*	Native			
				pSEV1	*tipA*p	*S. lividans*	Native			
	Streptavidin	*S. avidinii* NBRC13429	*S. lividans* 1,326	pTONA4	*pld*p	*S. cinnamoneus*	*pld*	*S. cinnamoneus*	-	Noda et al. (2015)
							Native			

The most frequently used approach for improved protein production is to increase the gene expression level, mainly by adopting strong promoters. One of the frequently used promoters is ermE*, which has been widely adopted as a strong constitutive promoter in *Streptomyces* (Bibb et al., 1985)*.* While many of the promoters from relatively close species are functional in the production host, regulatory elements for the promoters would also be conserved, resulting in transcriptional inhibition by negative regulators. To overcome this drawback, the promoter sequence or *Streptomyces* host was engineered to avoid negative regulation. For example, the activity of the strong inducible promoter *xysA*p from *S. halstedii* was further increased by deleting the homologs of negative regulators, BxlR and XlnR, in *S. lividans*, resulting in up to 70% higher production of a heterologous protein (Sevillano et al., 2016)*.* Another example is the production of chitosanase from *Kitasatospora* sp. N106, an actinomycete in *S. lividans* (Dubeau et al., 2011). To improve protein production, the negative regulatory gene *csnR* was deleted and/or two base pairs of the palindromic negative regulatory sequences in the promoter were mutated. The approach for increased gene expression level still holds more potential for improvement, since only a small number of promoters have been exploited (Table 5). Accumulation of transcriptomic data enables the identification and utilization of strong endogenous promoters, and a synthetic promoter library has been demonstrated for *Streptomyces* (Wang et al., 2013; Luo et al., 2015)*.* In addition to transcription activation-related factors, transcriptional terminators are worth investigating to improve gene expression. *Streptomyces* lacks an extremely strong expression system, such as the T7 expression system of *E. coli* BL21 (DE3) (although T7 RNA polymerase has been demonstrated in *S. lividans*); increasing the half-life of transcripts by exploiting strong transcriptional terminators may serve as an efficient tool for higher gene expression (Lussier et al., 2010; Lee et al., 2019; Hwang et al., 2021).

Enhancing protein secretion efficiency is another important approach that drastically increases the protein yield. The native signal peptides are generally functional for protein secretion in the *Streptomyces* host; however, optimization of signal peptides improves protein production and secretion in terms of both the proportion of secretion and product yield (Snajder et al., 2019). For example, various signal peptides were tested for the production of XylE, the catechol 2,3-dioxygenase, from *Pseudomonas putida* in *S. rimosus*, and extracellular XylE activity was highest when the lipase signal peptide of *S. rimosus* was utilized, while the *xysA* signal peptide of *S. halstedii* resulted in the highest secretion efficiency (Carrillo Rincon et al., 2018). In addition to examining diverse signal peptides, mutagenesis of signal peptides may elicit increased protein secretion. For example, the effect of charge variation in the α-amylase signal peptide of *S. venezuelae* on the secretion of mouse tumor necrosis factor α (mTNF-α) was investigated, and the introduction of one extra positive charge led to approximately 6.25-fold increased secretive production compared to the wild-type signal peptide (Lammertyn et al., 1998). Furthermore, when adopting a non-native signal peptide, introducing additional amino acids to the N-terminus of the mature protein to maintain the environment of the signal peptide cleavage site can improve production yield (Sevillano et al., 2016). Despite the effectiveness of signal peptide optimization, it is tedious to identify the proper signal peptide, since the secretion efficiency of each signal peptide differs for every target protein (Lammertyn et al., 1998; Carrillo Rincon et al., 2018). For a more general solution, modulating the secretion pathway, rather than diversifying signal peptides, has been demonstrated. Deletion of the *sipY* gene, which encodes a major signal peptidase, led to increased heterologous production of agarase from *S. coelicolor* in *S. lividans* (Gabarró et al., 2017)*.* The secretion of agarase in the SipY-deficient strain can be complemented by other signal peptidases, such as SipW, SipX, and SipZ, while extracellular protease activity is reduced (Parro et al., 1999; Escutia et al., 2006). Although the agarase of *S. coelicolor* is the only example of production improvement utilizing the SipY-deficient strain compared to the wild-type strain, the low extracellular protease activity would be favorable for the secretion of other proteins, and deletion of extracellular proteases may further elicit an increase in protein production.

In addition to increasing gene expression and optimizing the secretion system, cell morphology is related to protein secretion, and formation of clumps during culture is generally unfavorable. For example, overexpression of the *ssgA* gene, which is related to cell division and morphogenesis, led to improved protein yield. For other bacteria, codon optimization may be necessary for successful protein production. The genome of Streptomycetes is GC-rich (approximately 70%), and thus, GC-rich codons are preferred (Ruckert et al., 2015). In particular, the use of the rare leucine codon TTA would have to be avoided, since the codon is decoded by a dedicated tRNA species encoded by the *bldA* gene, and *bldA* is temporally regulated (Hesketh et al., 2007). For this reason, even proteins of *Streptomyces* origin are often codon-optimized to eliminate TTA when the production host is *Streptomyces* (Guan et al., 2015)*.*

## Perspectives on Future Engineering Approaches for Heterologous Protein Expression in *Streptomyces*

Although rational engineering approaches to improve the yield of heterologous production of recombinant proteins in *Streptomyces* have been introduced as described above, some challenges remain. First, genetic tools should be more efficient for cloning large BGCs (Nah et al., 2017). Technical advances in the preparation of intact large-size BGC sequences for *in vitro* cloning are needed, which may be accomplished by the optimization of genomic DNA extraction to minimize shearing. In addition, the improvement of TAR cloning efficiency in terms of a large number of small- or medium-sized BGC fragments should be considered. The development of a new bacterial TAR cloning hosts, instead of yeast, may be one of the solutions. Alternatively, each fragment can be *de novo* synthesized by a gene synthesis technique, which is beneficial for refactoring the standardized genetic parts, but may pose an issue with respect to length. BGC expression by the integrative vector system seems to be more stable and controllable than the replicative vector system. Comparative studies of integrative attachment sites and their genomic positions would assist in the optimization of BGC expression. Moreover, available synthetic parts for *Streptomyces*, including promoters, RBSs, riboswitches, and terminators for refactoring BGC genes should be expanded to tightly control gene expression in terms of strength and timing.

To determine the performance of expression systems and select the optimal clone with high yield among the libraries, high-throughput screening techniques are indispensable. Fluorescence-based reporter genes, such as the superfolder green fluorescent protein (*sfGFP*), have been applied in *Streptomyces*, which can lead to high-throughput screening of clones using fluorescence-activated cell sorting (FACS) (Bai et al., 2015). *Streptomyces* based cell-free protein synthesis (CFPS) is also noteworthy for high-throughput screening. Efforts have been made to improve CFPS systems (Li et al., 2017a; Moore et al., 2021). Since DNA can be directly added to the production environment, extremely large BGCs can be expressed easily compared to *in vivo* systems, owing to the low transformation efficiency of large-sized DNA. In addition, it can be performed on a multi-well plate scale that can easily facilitate the automation device application.

The main challenge in yield improvement by rational engineering after heterologous expression of BGC in *Streptomyces* is the lack of genetic information. In other words, finding engineering targets when the yield of heterologous expression is low is not simple. This is because heterologous BGC may interact with complex endogenous factors in the host which is the unpredictable interference hindering the orthogonal heterologous expression system (Beites and Mendes, 2015). The effects of heterologous host factors on the native host expression elements may be predicted by homology-based search, and it was the most common approach used in previous studies. However, BGC expression in the heterologous host phylogenetically close to the native host is not always better than that in the distant host. For example, a heterologous expression study of violacein BGC from *Pseudoalteromonas luteoviolacea* 2ta16 in the three different hosts revealed that violacein yield was higher in the phylogenetically distant host, *Agrobacterium tumefaciens* LBA4404, than in *E. coli,* because of the difference between PviR activator homologs (Zhang et al., 2017a)*.* Therefore, high-throughput approaches using systems and synthetic biology to design, build, and test all possible individual factors, followed by learning from the data for the positive feedback to the next DBTL cycle would be an effective strategy.

**FIGURE 2:**
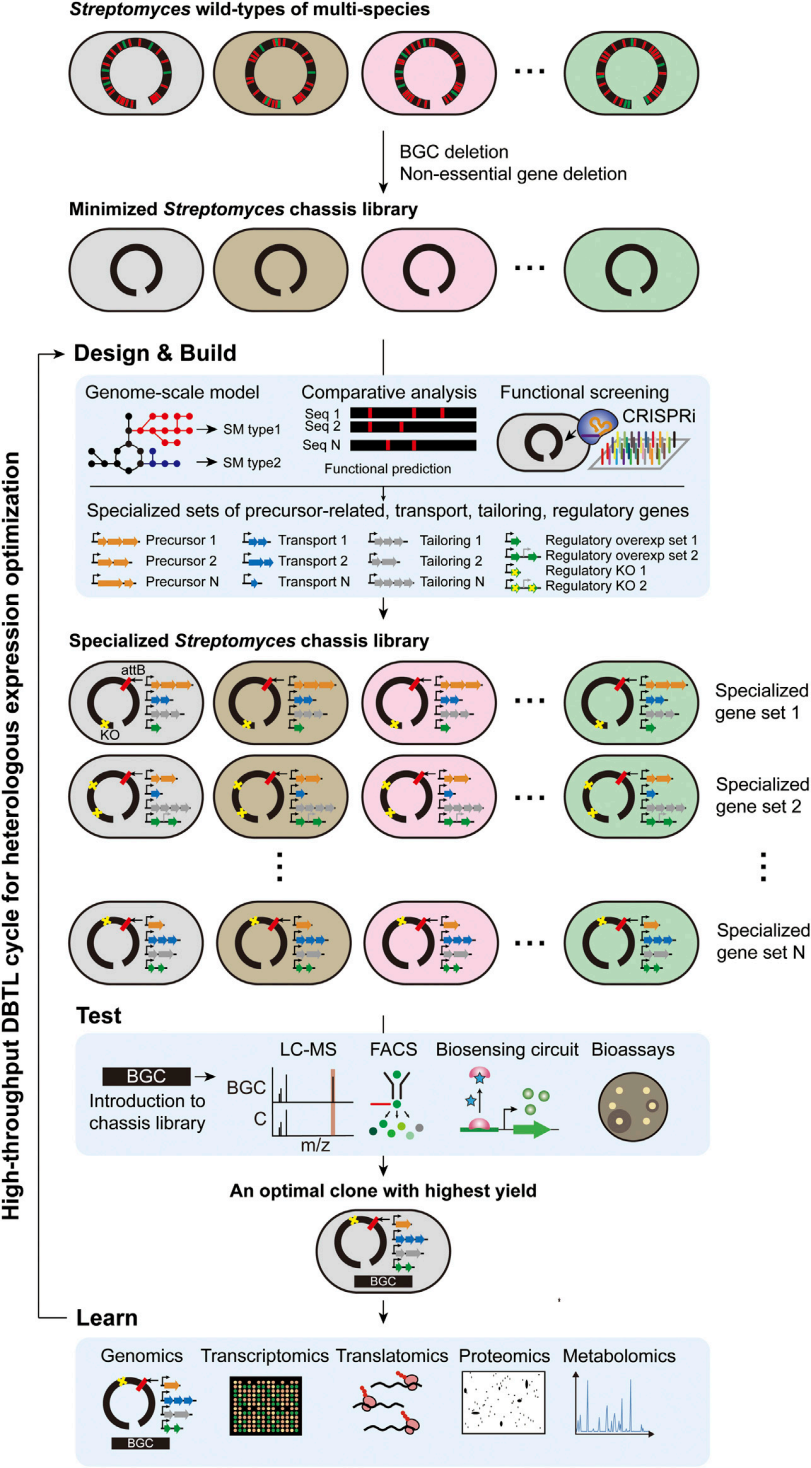
High-throughput DBTL cycle of rational engineering for heterologous production using specialized *Streptomyces* chassis library.

To realize a high-throughput DBTL approach, an optimal *Streptomyces* chassis system is urgently needed. However, it has been suggested that the “universal chassis” is difficult to be constructed because individual host factors differentially affect the expression of each BGC and protein of interest (Liu et al., 2018a; Ke and Yoshikuni, 2020). As introduced in the chassis development section above, *Streptomyces* chassis with reduced genome such as *S. coelicolor* M1146, *S. avermitilis* SUKA, *S. lividans* ΔYA9, and *S. albus* Del14 are generally efficient for the heterologous expression of BGCs due to their genome simplicity, but certain BGCs resulted in different expression levels among them. For example, heterologous expression screening of the BAC library of *S. albus* subsp. *chlorinus* NRRL B-24108 resulted that some BGCs were expressed in only one of *S. albus* Del14 and *S. lividans* ΔYA9 (Ahmed et al., 2020). For another example, the heterologous production yields of cephamycin C BGC of *S. clavuligerus* in *S. coelicolor* M1146 and *S. avermitilis* SUKA22 were both lower than the yield of the native host (Komatsu et al., 2013; Martinez-Burgo et al., 2014). The remaining biological complexity of these strains are likely to hamper them to be the “universal chassis” (Beites and Mendes, 2015). Instead, a “specialized chassis library” could be an alternative option. As a proof of concept, a “specialized *Streptomyces* chassis library” is demonstrated in this review (Figure 2). Several *Streptomyces* species previously used for heterologous hosts due to their general and specific advantages would be the starting strains, and their genomes will be minimized by removing all of their native BGCs and genes with negative effects on heterologous expression, such as insertion sequence (IS) elements, resulting in the “minimized *Streptomyces* chassis library.” These strains are expected to have robust growth, sufficient cellular energy, clear metabolic profiles, and genetic stability. Then, the combination of additional genes that govern precursor supply, transport, tailoring, and regulations will be determined, and each combination will be introduced to the minimized chassis library, resulting in the “specialized *Streptomyces* chassis library.” Precursor supply gene sets for specific BGC types might be predicted by a pan-genome model containing all biosynthetic reactions of known SMs, which integrates the information of all genome-scale models of reported *Streptomyces* species. For example, the biosynthetic gene sets for representative PK precursors, such as malonyl-CoA, methylmalonyl-CoA, ethylmalonyl-CoA, and methoxymalonyl-ACP, will be co-expressed in minimal chassis, resulting in a PK-specialized chassis. Accessory gene sets, including transport, resistance and tailoring genes, might be determined from their specificity, but related studies on this are scarce. Regulatory gene sets might be determined by high-throughput clustered regularly interspaced short palindromic repeats interference (CRISPRi) library-based approaches to screen regulatory genes with positive or negative effects on the expression of each BGC. As a result, the best clone with the highest yield of each BGC could be selected by introducing BGCs to the “specialized *Streptomyces* chassis library” and screening in a high-throughput manner. Learning from the systematic analysis of the best clone will aid in continuously optimizing the rational engineering design to improve the yield. This “specialized *Streptomyces* chassis library” will also be appropriate for testing uncharacterized BGCs to discover novel SMs.

As the production of recombinant proteins is less diverse than that of BGC products, the development of an optimal *Streptomyces* recombinant protein chassis may be a better choice than constructing a chassis library. Possible considerations of this chassis include the secretion pathway as the yield of functional recombinant proteins by either the Sec-pathway or Tat-pathway may be different according to their folding nature. In addition, comparative analysis of signal peptides may aid in determining the specific peptidases and controlling their expression for yield improvement. Also, more empirically, the further development of downstream processes such as purification steps will aid the improvement of the recombinant protein yield (Tripathi and Shrivastava, 2019). Although *S. lividans* was mostly used as a heterologous host for recombinant proteins, other species were also screened for their ability because some proteins were not effectively produced in *S. lividans* compared to other strains.

## Conclusion

This review focuses on rational engineering examples and perspectives of heterologous expression of BGCs and recombinant proteins in *Streptomyces*. Heterologous expression is an effective strategy for overcoming the native host in terms of growth, ease of genetic manipulation, and production yield. *Streptomyces* is an attractive heterologous expression host for BGCs and recombinant protein genes because of its functional biosynthetic enzyme expression, substrate availability, secretion systems, and other accessory genes. Rational engineering approaches for the yield improvement of heterologous expression in *Streptomyces* have been facilitated by the development of genetic tools, chassis construction, and additional genetic engineering strategies, emphasizing further demand for vigorous systems and synthetic biology approaches. Employing the high-throughput DBTL cycle using the “*Streptomyces* chassis library” or “*Streptomyces* chassis” for heterologous expression will open new horizons, expanding the availability and diversity of SMs and recombinant proteins.

## Data Availability

The original contributions presented in the study are included in the article/Supplementary Material, further inquiries can be directed to the corresponding author.

## References

[B1] Ahmed Y. Rebets Y. Estévez M. R. Zapp J. Myronovskyi M. Luzhetskyy A. (2020). Engineering of Streptomyces Lividans for Heterologous Expression of Secondary Metabolite Gene Clusters. Microb. Cel Fact 19, 5. 10.1186/s12934-020-1277-8 PMC6950998 31918711

[B2] Alexander D. C. Rock J. He X. Brian P. Miao V. Baltz R. H. (2010). Development of a Genetic System for Combinatorial Biosynthesis of Lipopeptides in Streptomyces Fradiae and Heterologous Expression of the A54145 Biosynthesis Gene Cluster. Appl. Environ. Microbiol. 76, 6877–6887. 10.1128/aem.01248-10 20802082 PMC2953024

[B3] Anné J. Vrancken K. Van Mellaert L. Van Impe J. Bernaerts K. (2014). Protein Secretion Biotechnology in Gram-Positive Bacteria with Special Emphasis on Streptomyces Lividans. Biochim. Biophys. Acta (Bba) - Mol. Cel Res. 1843, 1750–1761. 10.1016/j.bbamcr.2013.12.023 24412306

[B4] Aubry C. Pernodet J. L. Lautru S. (2019). Modular and Integrative Vectors for Synthetic Biology Applications in Streptomyces Spp. Appl. Environ. Microbiol. 85. 10.1128/AEM.00485-19 PMC6677859 31175189

[B5] Bai C. Zhang Y. Zhao X. Hu Y. Xiang S. Miao J. (2015). Exploiting a Precise Design of Universal Synthetic Modular Regulatory Elements to Unlock the Microbial Natural Products in Streptomyces. Proc. Natl. Acad. Sci. USA 112, 12181–12186. 10.1073/pnas.1511027112 26374838 PMC4593075

[B6] Baltz R. H. (2012). Streptomyces Temperate Bacteriophage Integration Systems for Stable Genetic Engineering of Actinomycetes (And Other Organisms). J. Ind. Microbiol. Biotechnol. 39, 661–672. 10.1007/s10295-011-1069-6 22160317

[B7] Barbuto Ferraiuolo S. Cammarota M. Schiraldi C. Restaino O. F. (2021). Streptomycetes as Platform for Biotechnological Production Processes of Drugs. Appl. Microbiol. Biotechnol. 105, 551–568. 10.1007/s00253-020-11064-2 33394149 PMC7780072

[B8] Behnken S. Lincke T. Kloss F. Ishida K. Hertweck C. (2012). Antiterminator-mediated Unveiling of Cryptic Polythioamides in an Anaerobic Bacterium. Angew. Chem. Int. Ed. 51, 2425–2428. 10.1002/anie.201108214 22287490

[B9] Beites T. Mendes M. V. (2015). Chassis Optimization as a Cornerstone for the Application of Synthetic Biology Based Strategies in Microbial Secondary Metabolism. Front. Microbiol. 6, 906. 10.3389/fmicb.2015.00906 26441855 PMC4563238

[B10] Bekiesch P. Basitta P. Apel A. K. (2016). Challenges in the Heterologous Production of Antibiotics inStreptomyces. Arch. Pharm. Chem. Life Sci. 349, 594–601. 10.1002/ardp.201600058 27258165

[B11] Bentley S. D. Chater K. F. Cerdeño-Tárraga A.-M. Challis G. L. Thomson N. R. James K. D. (2002). Complete Genome Sequence of the Model Actinomycete Streptomyces Coelicolor A3(2). Nature 417, 141–147. 10.1038/417141a 12000953

[B12] Berini F. Casartelli M. Montali A. Reguzzoni M. Tettamanti G. Marinelli F. (2019). Metagenome-Sourced Microbial Chitinases as Potential Insecticide Proteins. Front. Microbiol. 10, 1358. 10.3389/fmicb.2019.01358 31275279 PMC6591435

[B13] Berini F. Marinelli F. Binda E. (2020). Streptomycetes: Attractive Hosts for Recombinant Protein Production. Front. Microbiol. 11, 1958. 10.3389/fmicb.2020.01958 32973711 PMC7468451

[B14] Bibb M. J. Janssen G. R. Ward J. M. (1985). Cloning and Analysis of the Promoter Region of the Erythromycin Resistance Gene (ermE) of Streptomyces Erythraeus. Gene 38, 215–226. 10.1016/0378-1119(85)90220-3 2998943

[B15] Bilyk B. Luzhetskyy A. (2014). Unusual Site-specific DNA Integration into the Highly Active Pseudo-attB of the Streptomyces Albus J1074 Genome. Appl. Microbiol. Biotechnol. 98, 5095–5104. 10.1007/s00253-014-5605-y 24566921

[B16] Bilyk O. Sekurova O. N. Zotchev S. B. Luzhetskyy A. (2016). Cloning and Heterologous Expression of the Grecocycline Biosynthetic Gene Cluster. PLoS One 11, e0158682. 10.1371/journal.pone.0158682 27410036 PMC4943663

[B17] Bonet B. Teufel R. Crüsemann M. Ziemert N. Moore B. S. (2015). Direct Capture and Heterologous Expression of Salinispora Natural Product Genes for the Biosynthesis of Enterocin. J. Nat. Prod. 78, 539–542. 10.1021/np500664q 25382643 PMC4380194

[B18] Bu Q.-T. Yu P. Wang J. Li Z.-Y. Chen X.-A. Mao X.-M. (2019). Rational Construction of Genome-Reduced and High-Efficient Industrial Streptomyces Chassis Based on Multiple Comparative Genomic Approaches. Microb. Cel Fact 18, 16. 10.1186/s12934-019-1055-7 PMC6348691 30691531

[B19] Cai W. Zhang W. (2018). Engineering Modular Polyketide Synthases for Production of Biofuels and Industrial Chemicals. Curr. Opin. Biotechnol. 50, 32–38. 10.1016/j.copbio.2017.08.017 28946011 PMC5862724

[B20] Carrillo Rincón A. F. Magdevska V. Kranjc L. Fujs Š. Müller R. Petković H. (2018). Production of Extracellular Heterologous Proteins in Streptomyces Rimosus, Producer of the Antibiotic Oxytetracycline. Appl. Microbiol. Biotechnol. 102, 2607–2620. 10.1007/s00253-018-8793-z 29417200

[B21] Cavaletti L. Taravella A. Carrano L. Carenzi G. Sigurtà A. Solinas N. (2019). E40, a Novel Microbial Protease Efficiently Detoxifying Gluten Proteins, for the Dietary Management of Gluten Intolerance. Sci. Rep. 9, 13147. 10.1038/s41598-019-48299-7 31511534 PMC6739405

[B22] Chiu M. L. Folcher M. Katoh T. Puglia A. M. Vohradsky J. Yun B.-S. (1999). Broad Spectrum Thiopeptide Recognition Specificity of theStreptomyces Lividans TipAL Protein and its Role in Regulating Gene Expression. J. Biol. Chem. 274, 20578–20586. 10.1074/jbc.274.29.20578 10400688

[B23] Côté A. Shareck F. (2010). Expression and Characterization of a Novel Heterologous Moderately Thermostable Lipase Derived from Metagenomics in Streptomyces Lividans. J. Ind. Microbiol. Biotechnol. 37, 883–891. 10.1007/s10295-010-0735-4 20495942

[B24] Crawford D. L. (1978). Lignocellulose Decomposition by Selected Streptomyces Strains. Appl. Environ. Microbiol. 35, 1041–1045. 10.1128/aem.35.6.1041-1045.1978 677871 PMC242982

[B25] D'agostino P. M. Gulder T. A. M. (2018). Direct Pathway Cloning Combined with Sequence- and Ligation-independent Cloning for Fast Biosynthetic Gene Cluster Refactoring and Heterologous Expression. ACS Synth. Biol. 7, 1702–1708. 10.1021/acssynbio.8b00151 29940102

[B26] Du D. Wang L. Tian Y. Liu H. Tan H. Niu G. (2015). Genome Engineering and Direct Cloning of Antibiotic Gene Clusters via Phage ϕBT1 Integrase-Mediated Site-specific Recombination in Streptomyces. Sci. Rep. 5, 8740. 10.1038/srep08740 25737113 PMC4349145

[B27] Dubeau M.-P. Guay I. Brzezinski R. (2011). Modification of Genetic Regulation of a Heterologous Chitosanase Gene in Streptomyces Lividans TK24 Leads to Chitosanase Production in the Absence of Chitosan. Microb. Cel Fact 10, 7. 10.1186/1475-2859-10-7 PMC3048496 21310076

[B28] Edgar R. Bibi E. (1997). MdfA, an *Escherichia coli* Multidrug Resistance Protein with an Extraordinarily Broad Spectrum of Drug Recognition. J. Bacteriol. 179, 2274–2280. 10.1128/jb.179.7.2274-2280.1997 9079913 PMC178964

[B29] Escutia M. R. Val G. Palacín A. Geukens N. Anné J. Mellado R. P. (2006). Compensatory Effect of the minorStreptomyces Lividans Type I Signal Peptidases on the SipY Major Signal Peptidase Deficiency as Determined by Extracellular Proteome Analysis. Proteomics 6, 4137–4146. 10.1002/pmic.200500927 16786486

[B30] Eyles T. H. Vior N. M. Truman A. W. (2018). Rapid and Robust Yeast-Mediated Pathway Refactoring Generates Multiple New Bottromycin-Related Metabolites. ACS Synth. Biol. 7, 1211–1218. 10.1021/acssynbio.8b00038 29694038

[B31] Fan K. Pan G. Peng X. Zheng J. Gao W. Wang J. (2012). Identification of JadG as the B Ring Opening Oxygenase Catalyzing the Oxidative C-C Bond Cleavage Reaction in Jadomycin Biosynthesis. Chem. Biol. 19, 1381–1390. 10.1016/j.chembiol.2012.09.009 23177193

[B32] Farkasovska J. Godany A. (2012). Analysis of the Site-specific Integration System of the Streptomyces Aureofaciens Phage Mu1/6. Curr. Microbiol. 64, 226–233. 22143397 10.1007/s00284-011-0054-7

[B33] Fayed B. Younger E. Taylor G. Smith M. C. M. (2014). A Novel Streptomyces Spp. Integration Vector Derived from the S. Venezuelaephage, SV1. BMC Biotechnol. 14, 51. 10.1186/1472-6750-14-51 24885867 PMC4068962

[B34] Feilmeier B. J. Iseminger G. Schroeder D. Webber H. Phillips G. J. (2000). Green Fluorescent Protein Functions as a Reporter for Protein Localization in *Escherichia coli* . J. Bacteriol. 182, 4068–4076. 10.1128/jb.182.14.4068-4076.2000 10869087 PMC94594

[B35] Feng Z. Wang L. Rajski S. R. Xu Z. Coeffet-Legal M. F. Shen B. (2009). Engineered Production of Iso-Migrastatin in Heterologous Streptomyces Hosts. Bioorg. Med. Chem. 17, 2147–2153. 10.1016/j.bmc.2008.10.074 19010685 PMC3075207

[B36] Fisher A. C. Kim J.-Y. Perez-Rodriguez R. Tullman-Ercek D. Fish W. R. Henderson L. A. (2008). Exploration of Twin-Arginine Translocation for Expression and Purification of Correctly Folded Proteins inEscherichia Coli. Microb. Biotechnol. 1, 403–415. 10.1111/j.1751-7915.2008.00041.x 21261860 PMC3057487

[B37] Fogg P. C. M. Haley J. A. Stark W. M. Smith M. C. M. (2017). Genome Integration and Excision by a New Streptomyces Bacteriophage, ϕJoe. Appl. Environ. Microbiol. 83, e02767. 10.1128/AEM.02767-16 28003200 PMC5311408

[B38] Fong R. Hu Z. Hutchinson C. R. Huang J. Cohen S. Kao C. (2007). Characterization of a Large, Stable, High-Copy-Number Streptomyces Plasmid that Requires Stability and Transfer Functions for Heterologous Polyketide Overproduction. Appl. Environ. Microbiol. 73, 1296–1307. 10.1128/aem.01888-06 17142363 PMC1828658

[B39] Foor F. Roberts G. P. Morin N. Snyder L. Hwang M. Gibbons P. H. (1985). Isolation and Characterization of the Streptomyces Cattleya Temperate Phage TG1. Gene 39, 11–16. 10.1016/0378-1119(85)90101-5 3000891

[B40] Fu J. Bian X. Hu S. Wang H. Huang F. Seibert P. M. (2012). Full-length RecE Enhances Linear-Linear Homologous Recombination and Facilitates Direct Cloning for Bioprospecting. Nat. Biotechnol. 30, 440–446. 10.1038/nbt.2183 22544021

[B41] Gabarró M. L. V. Gullón S. Vicente R. L. Caminal G. Mellado R. P. López-Santín J. (2017). A Streptomyces Lividans SipY Deficient Strain as a Host for Protein Production: Standardization of Operational Alternatives for Model Proteins. J. Chem. Technology Biotechnol. 92, 217–223. 10.1002/jctb.4933

[B42] Gamboa-Suasnavart R. A. Valdez-Cruz N. A. Cordova-Davalos L. E. Martinez-Sotelo J. A. Servin-Gonzalez L. Espitia C. (2011). The O-Mannosylation and Production of Recombinant APA (45/47 KDa) Protein from *Mycobacterium tuberculosis* in Streptomyces Lividans Is Affected by Culture Conditions in Shake Flasks. Microb. Cel Fact 10, 110. 10.1186/1475-2859-10-110 PMC3266650 22185589

[B43] Gao H. Liu M. Zhou X. Liu J. Zhuo Y. Gou Z. (2010). Identification of Avermectin-High-Producing Strains by High-Throughput Screening Methods. Appl. Microbiol. Biotechnol. 85, 1219–1225. 10.1007/s00253-009-2345-5 19957083

[B44] Ghosh P. Wasil L. R. Hatfull G. F. (2006). Control of Phage Bxb1 Excision by a Novel Recombination Directionality Factor. Plos Biol. 4, e186. 10.1371/journal.pbio.0040186 16719562 PMC1470463

[B45] Gibson D. G. Young L. Chuang R. Y. Venter J. C. Hutchison C. A. 3rd Smith H. O. (2009). Enzymatic Assembly of DNA Molecules up to Several Hundred Kilobases. Nat. Methods 6, 343–345. 10.1038/nmeth.1318 19363495

[B46] Gomez-Escribano J. P. Bibb M. J. (2011). Engineering Streptomyces Coelicolor for Heterologous Expression of Secondary Metabolite Gene Clusters. Microb. Biotechnol. 4, 207–215. 10.1111/j.1751-7915.2010.00219.x 21342466 PMC3818861

[B47] Gomez-Escribano J. P. Bibb M. J. (2014). Heterologous Expression of Natural Product Biosynthetic Gene Clusters in Streptomyces Coelicolor: from Genome Mining to Manipulation of Biosynthetic Pathways. J. Ind. Microbiol. Biotechnol. 41, 425–431. 10.1007/s10295-013-1348-5 24096958

[B48] Gregory M. A. Till R. Smith M. C. (2003). Integration Site for Streptomyces Phage phiBT1 and Development of Site-specific Integrating Vectors. J. Bacteriol. 185, 5320–5323. 10.1128/jb.185.17.5320-5323.2003 12923110 PMC180994

[B49] Greunke C. Duell E. R. D'agostino P. M. Glockle A. Lamm K. Gulder T. A. M. (2018). Direct Pathway Cloning (DiPaC) to Unlock Natural Product Biosynthetic Potential. Metab. Eng. 47, 334–345. 10.1016/j.ymben.2018.03.010 29548983

[B50] Guan C. Cui W. He X. Hu X. Xu J. Du G. (2015). Construction and Development of a Novel Expression System of Streptomyces. Protein Expr. Purif. 113, 17–22. 10.1016/j.pep.2015.04.009 25956536

[B51] Gullon S. Marin S. Mellado R. P. (2015a). Overproduction of a Model Sec- and Tat-dependent Secretory Protein Elicits Different Cellular Responses in Streptomyces Lividans. PLoS One 10, e0133645. 10.1371/journal.pone.0133645 26200356 PMC4511581

[B52] Gullon S. Olano C. Abdelfattah M. S. Brana A. F. Rohr J. Mendez C. (2006). Isolation, Characterization, and Heterologous Expression of the Biosynthesis Gene Cluster for the Antitumor Anthracycline Steffimycin. Appl. Environ. Microbiol. 72, 4172–4183. 10.1128/aem.00734-06 16751529 PMC1489666

[B53] Gullon S. Vicente R. L. Valverde J. R. Marin S. Mellado R. P. (2015b). Exploring the Feasibility of the Sec Route to Secrete Proteins Using the Tat Route in Streptomyces Lividans. Mol. Biotechnol. 57, 931–938. 10.1007/s12033-015-9883-0 26202494

[B54] Hamed M. B. Anne J. Karamanou S. Economou A. (2018). Streptomyces Protein Secretion and its Application in Biotechnology. FEMS Microbiol. Lett. 365, 1. 10.1093/femsle/fny250 30299471

[B55] Hamed M. B. Karamanou S. Olafsdottir S. Basilio J. S. M. Simoens K. Tsolis K. C. (2017). Large-scale Production of a Thermostable Rhodothermus Marinus Cellulase by Heterologous Secretion from Streptomyces Lividans. Microb. Cel Fact 16, 232. 10.1186/s12934-017-0847-x PMC5741968 29274637

[B56] Harvey C. J. B. Tang M. Schlecht U. Horecka J. Fischer C. R. Lin H. C. (2018). HEx: A Heterologous Expression Platform for the Discovery of Fungal Natural Products. Sci. Adv. 4, eaar5459. 10.1126/sciadv.aar5459 29651464 PMC5895447

[B57] Heidorn T. Camsund D. Huang H. H. Lindberg P. Oliveira P. Stensjo K. (2011). Synthetic Biology in Cyanobacteria Engineering and Analyzing Novel Functions. Methods Enzymol. 497, 539–579. 10.1016/b978-0-12-385075-1.00024-x 21601103

[B58] Hesketh A. Bucca G. Laing E. Flett F. Hotchkiss G. Smith C. P. (2007). New Pleiotropic Effects of Eliminating a Rare tRNA from Streptomyces Coelicolor, Revealed by Combined Proteomic and Transcriptomic Analysis of Liquid Cultures. BMC Genomics 8, 261. 10.1186/1471-2164-8-261 17678549 PMC2000904

[B59] Hong H. J. Hutchings M. I. Hill L. M. Buttner M. J. (2005). The Role of the Novel Fem Protein VanK in Vancomycin Resistance in Streptomyces Coelicolor. J. Biol. Chem. 280, 13055–13061. 10.1074/jbc.m413801200 15632111

[B60] Hong J. S. Park S. H. Choi C. Y. Sohng J. K. Yoon Y. J. (2004). New Olivosyl Derivatives of Methymycin/pikromycin from an Engineered Strain of Streptomyces Venezuelae. FEMS Microbiol. Lett. 238, 391–399. 10.1111/j.1574-6968.2004.tb09781.x 15358425

[B61] Horbal L. Fedorenko V. Luzhetskyy A. (2014). Novel and Tightly Regulated Resorcinol and Cumate-Inducible Expression Systems for Streptomyces and Other Actinobacteria. Appl. Microbiol. Biotechnol. 98, 8641–8655. 10.1007/s00253-014-5918-x 25012786

[B62] Horbal L. Marques F. Nadmid S. Mendes M. V. Luzhetskyy A. (2018a). Secondary Metabolites Overproduction through Transcriptional Gene Cluster Refactoring. Metab. Eng. 49, 299–315. 10.1016/j.ymben.2018.09.010 30240601

[B63] Horbal L. Siegl T. Luzhetskyy A. (2018b). A Set of Synthetic Versatile Genetic Control Elements for the Efficient Expression of Genes in Actinobacteria. Sci. Rep. 8, 491. 10.1038/s41598-017-18846-1 29323285 PMC5765039

[B64] Hu H. Zhang Q. Ochi K. (2002). Activation of Antibiotic Biosynthesis by Specified Mutations in the rpoB Gene (Encoding the RNA Polymerase Beta Subunit) of Streptomyces Lividans. J. Bacteriol. 184, 3984–3991. 10.1128/jb.184.14.3984-3991.2002 PMC135172 12081971

[B65] Huang H. Zheng G. Jiang W. Hu H. Lu Y. (2015). One-step High-Efficiency CRISPR/Cas9-mediated Genome Editing in Streptomyces. Acta Biochim. Biophys. Sin (Shanghai) 47, 231–243. 10.1093/abbs/gmv007 25739462

[B66] Huang J. Shi J. Molle V. Sohlberg B. Weaver D. Bibb M. J. (2005). Cross-regulation Among Disparate Antibiotic Biosynthetic Pathways of Streptomyces Coelicolor. Mol. Microbiol. 58, 1276–1287. 10.1111/j.1365-2958.2005.04879.x 16313616

[B67] Huff J. Czyz A. Landick R. Niederweis M. (2010). Taking Phage Integration to the Next Level as a Genetic Tool for Mycobacteria. Gene 468, 8–19. 10.1016/j.gene.2010.07.012 20692326 PMC2952446

[B68] Huo L. Hug J. J. Fu C. Bian X. Zhang Y. Muller R. (2019). Heterologous Expression of Bacterial Natural Product Biosynthetic Pathways. Nat. Prod. Rep. 36, 1412–1436. 10.1039/c8np00091c 30620035

[B69] Hwang S. Lee N. Cho S. Palsson B. Cho B. K. (2020). Repurposing Modular Polyketide Synthases and Non-ribosomal Peptide Synthetases for Novel Chemical Biosynthesis. Front. Mol. Biosci. 7, 87. 10.3389/fmolb.2020.00087 32500080 PMC7242659

[B70] Hwang S. Lee N. Choe D. Lee Y. Kim W. Jeong Y. (2021). Elucidating the Regulatory Elements for Transcription Termination and Posttranscriptional Processing in the Streptomyces Clavuligerus Genome. mSystems 6, 1. 10.1128/msystems.01013-20 PMC8269248 33947798

[B71] Jiang L. Wei J. Li L. Niu G. Tan H. (2013). Combined Gene Cluster Engineering and Precursor Feeding to Improve Gougerotin Production in Streptomyces Graminearus. Appl. Microbiol. Biotechnol. 97, 10469–10477. 10.1007/s00253-013-5270-6 24121866

[B72] Jiang W. Zhao X. Gabrieli T. Lou C. Ebenstein Y. Zhu T. F. (2015). Cas9-Assisted Targeting of CHromosome Segments CATCH Enables One-step Targeted Cloning of Large Gene Clusters. Nat. Commun. 6, 8101. 10.1038/ncomms9101 26323354 PMC4569707

[B73] Jiang W. Zhu T. F. (2016). Targeted Isolation and Cloning of 100-kb Microbial Genomic Sequences by Cas9-Assisted Targeting of Chromosome Segments. Nat. Protoc. 11, 960–975. 10.1038/nprot.2016.055 27101517

[B74] Jones A. C. Gust B. Kulik A. Heide L. Buttner M. J. Bibb M. J. (2013). Phage P1-Derived Artificial Chromosomes Facilitate Heterologous Expression of the FK506 Gene Cluster. PLoS One 8, e69319. 10.1371/journal.pone.0069319 23874942 PMC3708917

[B75] Joska T. M. Mashruwala A. Boyd J. M. Belden W. J. (2014). A Universal Cloning Method Based on Yeast Homologous Recombination that Is Simple, Efficient, and Versatile. J. Microbiol. Methods 100, 46–51. 10.1016/j.mimet.2013.11.013 24418681 PMC4521215

[B76] Jung W. S. Han A. R. Hong J. S. Park S. R. Choi C. Y. Park J. W. (2007). Bioconversion of 12-, 14-, and 16-membered Ring Aglycones to Glycosylated Macrolides in an Engineered Strain of Streptomyces Venezuelae. Appl. Microbiol. Biotechnol. 76, 1373–1381. 10.1007/s00253-007-1101-y 17665193

[B77] Jung W. S. Lee S. K. Hong J. S. Park S. R. Jeong S. J. Han A. R. (2006). Heterologous Expression of Tylosin Polyketide Synthase and Production of a Hybrid Bioactive Macrolide in Streptomyces Venezuelae. Appl. Microbiol. Biotechnol. 72, 763–769. 10.1007/s00253-006-0318-5 16493552

[B78] Kang H. S. Kim E. S. (2021). Recent Advances in Heterologous Expression of Natural Product Biosynthetic Gene Clusters in Streptomyces Hosts. Curr. Opin. Biotechnol. 69, 118–127. 10.1016/j.copbio.2020.12.016 33445072

[B79] Ke J. Yoshikuni Y. (2020). Multi-chassis Engineering for Heterologous Production of Microbial Natural Products. Curr. Opin. Biotechnol. 62, 88–97. 10.1016/j.copbio.2019.09.005 31639618

[B80] Khaleel T. Younger E. Mcewan A. R. Varghese A. S. Smith M. C. (2011). A Phage Protein that Binds phiC31 Integrase to Switch its Directionality. Mol. Microbiol. 80, 1450–1463. 10.1111/j.1365-2958.2011.07696.x 21564337

[B81] Kim E. J. Yang I. Yoon Y. J. (2015). Developing Streptomyces Venezuelae as a Cell Factory for the Production of Small Molecules Used in Drug Discovery. Arch. Pharm. Res. 38, 1606–1616. 10.1007/s12272-015-0638-z 26211662

[B82] Kim M. R. Choeng Y. H. Chi W. J. Kang D. K. Hong S. K. (2010). Heterologous Production of Streptokinase as a Secretory Form in Streptomyces Lividans and Nonsecretory Form in *Escherichia coli* . J. Microbiol. Biotechnol. 20, 132–137. 10.4014/jmb.0906.06005 20134244

[B83] Ko B. D'alessandro J. Douangkeomany L. Stumpf S. Debutts A. Blodgett J. (2020). Construction of a New Integrating Vector from Actinophage varphiOZJ and its Use in Multiplex Streptomyces Transformation. J. Ind. Microbiol. Biotechnol. 47, 73–81. 10.1007/s10295-019-02246-7 31705217

[B84] Komatsu M. Komatsu K. Koiwai H. Yamada Y. Kozone I. Izumikawa M. (2013). Engineered Streptomyces Avermitilis Host for Heterologous Expression of Biosynthetic Gene Cluster for Secondary Metabolites. ACS Synth. Biol. 2, 384–396. 10.1021/sb3001003 23654282 PMC3932656

[B85] Komatsu M. Uchiyama T. Omura S. Cane D. E. Ikeda H. (2010). Genome-minimized Streptomyces Host for the Heterologous Expression of Secondary Metabolism. Proc. Natl. Acad. Sci. U S A. 107, 2646–2651. 10.1073/pnas.0914833107 20133795 PMC2823899

[B86] Komeda H. Hori Y. Kobayashi M. Shimizu S. (1996). Transcriptional Regulation of the Rhodococcus Rhodochrous J1 nitA Gene Encoding a Nitrilase. Proc. Natl. Acad. Sci. U S A. 93, 10572–10577. 10.1073/pnas.93.20.10572 8855219 PMC38194

[B87] Kouprina N. Larionov V. (2006). TAR Cloning: Insights into Gene Function, Long-Range Haplotypes and Genome Structure and Evolution. Nat. Rev. Genet. 7, 805–812. 10.1038/nrg1943 16983376

[B88] Labes G. Bibb M. Wohlleben W. (1997). Isolation and Characterization of a strong Promoter Element from the Streptomyces Ghanaensis Phage I19 Using the Gentamicin Resistance Gene (aacC1) of Tn 1696 as Reporter. Microbiology (Reading) 143 (Pt 5), 1503–1512. 10.1099/00221287-143-5-1503 9168600

[B89] Lammertyn E. Desmyter S. Schacht S. Van Mellaert L. Anne J. (1998). Influence of Charge Variation in the Streptomyces Venezuelae Alpha-Amylase Signal Peptide on Heterologous Protein Production by Streptomyces Lividans. Appl. Microbiol. Biotechnol. 49, 424–430. 10.1007/s002530051193 9615485

[B90] Lee N. Hwang S. Kim W. Lee Y. Kim J. H. Cho S. (2021). Systems and Synthetic Biology to Elucidate Secondary Metabolite Biosynthetic Gene Clusters Encoded in Streptomyces Genomes. Nat. Prod. Rep. 38, 1330–1361. 10.1039/d0np00071j 33393961

[B91] Lee Y. Lee N. Jeong Y. Hwang S. Kim W. Cho S. (2019). The Transcription Unit Architecture of Streptomyces Lividans TK24. Front. Microbiol. 10, 2074. 10.3389/fmicb.2019.02074 31555254 PMC6742748

[B92] Li J. Chen D. Yu Z. Zhao L. Zhang R. (2013a). Improvement of Expression Level of Keratinase Sfp2 from Streptomyces Fradiae by Site-Directed Mutagenesis of its N-Terminal Pro-sequence. Biotechnol. Lett. 35, 743–749. 10.1007/s10529-013-1139-0 23355035

[B93] Li J. Wang H. Kwon Y. C. Jewett M. C. (2017a). Establishing a High Yielding Streptomyces-Based Cell-free Protein Synthesis System. Biotechnol. Bioeng. 114, 1343–1353. 10.1002/bit.26253 28112394

[B94] Li J. X. Zhao L. M. Wu R. J. Zheng Z. J. Zhang R. J. (2013b). High-level Overproduction of Thermobifida Enzyme in Streptomyces Lividans Using a Novel Expression Vector. Int. J. Mol. Sci. 14, 18629–18639. 10.3390/ijms140918629 24025422 PMC3794799

[B95] Li L. Jiang W. Lu Y. (2018). A Modified Gibson Assembly Method for Cloning Large DNA Fragments with High GC Contents. Methods Mol. Biol. 1671, 203–209. 10.1007/978-1-4939-7295-1_13 29170961

[B96] Li L. Zhao Y. Ruan L. Yang S. Ge M. Jiang W. (2015). A Stepwise Increase in Pristinamycin II Biosynthesis by Streptomyces Pristinaespiralis through Combinatorial Metabolic Engineering. Metab. Eng. 29, 12–25. 10.1016/j.ymben.2015.02.001 25708513

[B97] Li L. Zheng G. Chen J. Ge M. Jiang W. Lu Y. (2017b). Multiplexed Site-specific Genome Engineering for Overproducing Bioactive Secondary Metabolites in Actinomycetes. Metab. Eng. 40, 80–92. 10.1016/j.ymben.2017.01.004 28088540

[B98] Liew C. W. Nilsson M. Chen M. W. Sun H. Cornvik T. Liang Z. X. (2012). Crystal Structure of the Acyltransferase Domain of the Iterative Polyketide Synthase in Enediyne Biosynthesis. J. Biol. Chem. 287, 23203–23215. 10.1074/jbc.m112.362210 22589546 PMC3391151

[B99] Linares-Otoya L. Linares-Otoya V. Armas-Mantilla L. Blanco-Olano C. Crusemann M. Ganoza-Yupanqui M. L. (2017). Identification and Heterologous Expression of the Kocurin Biosynthetic Gene Cluster. Microbiology (Reading) 163, 1409–1414. 10.1099/mic.0.000538 28942758

[B100] Liu C. Zhang J. Lu C. Shen Y. (2015). Heterologous Expression of Galbonolide Biosynthetic Genes in Streptomyces Coelicolor. Antonie Van Leeuwenhoek 107, 1359–1366. 10.1007/s10482-015-0415-5 25735435

[B101] Liu H. Jiang H. Haltli B. Kulowski K. Muszynska E. Feng X. (2009). Rapid Cloning and Heterologous Expression of the Meridamycin Biosynthetic Gene Cluster Using a Versatile Escherichia Coli-Streptomyces Artificial Chromosome Vector, pSBAC. J. Nat. Prod. 72, 389–395. 10.1021/np8006149 19191550

[B102] Liu L. Yang H. Shin H. D. Li J. Du G. Chen J. (2013). Recent Advances in Recombinant Protein Expression by Corynebacterium, Brevibacterium, and Streptomyces: from Transcription and Translation Regulation to Secretion Pathway Selection. Appl. Microbiol. Biotechnol. 97, 9597–9608. 10.1007/s00253-013-5250-x 24068337

[B103] Liu R. Deng Z. Liu T. (2018a). Streptomyces Species: Ideal Chassis for Natural Product Discovery and Overproduction. Metab. Eng. 50, 74–84. 10.1016/j.ymben.2018.05.015 29852270

[B104] Liu S. Wang M. Du G. Chen J. (2016). Improving the Active Expression of Transglutaminase in Streptomyces Lividans by Promoter Engineering and Codon Optimization. BMC Biotechnol. 16, 75. 10.1186/s12896-016-0304-7 27793152 PMC5084433

[B105] Liu X. Liu D. Xu M. Tao M. Bai L. Deng Z. (2018b). Reconstitution of Kinamycin Biosynthesis within the Heterologous Host Streptomyces Albus J1074. J. Nat. Prod. 81, 72–77. 10.1021/acs.jnatprod.7b00652 29338229

[B106] Lombo F. Velasco A. Castro A. De La Calle F. Brana A. F. Sanchez-Puelles J. M. (2006). Deciphering the Biosynthesis Pathway of the Antitumor Thiocoraline from a marine Actinomycete and its Expression in Two Streptomyces Species. Chembiochem 7, 366–376. 10.1002/cbic.200500325 16408310

[B107] Lule I. Maldonado B. D'huys P. J. Van Mellaert L. Van Impe J. Bernaerts K. (2012). On the Influence of Overexpression of Phosphoenolpyruvate Carboxykinase in Streptomyces Lividans on Growth and Production of Human Tumour Necrosis Factor-Alpha. Appl. Microbiol. Biotechnol. 96, 367–372. 10.1007/s00253-012-4182-1 22797598

[B108] Luo Y. Enghiad B. Zhao H. (2016). New Tools for Reconstruction and Heterologous Expression of Natural Product Biosynthetic Gene Clusters. Nat. Prod. Rep. 33, 174–182. 10.1039/c5np00085h 26647833 PMC4742407

[B109] Luo Y. Huang H. Liang J. Wang M. Lu L. Shao Z. (2013). Activation and Characterization of a Cryptic Polycyclic Tetramate Macrolactam Biosynthetic Gene Cluster. Nat. Commun. 4, 2894. 10.1038/ncomms3894 24305602 PMC3969335

[B110] Luo Y. Zhang L. Barton K. W. Zhao H. (2015). Systematic Identification of a Panel of Strong Constitutive Promoters from Streptomyces Albus. ACS Synth. Biol. 4, 1001–1010. 10.1021/acssynbio.5b00016 25924180

[B111] Lussier F. X. Denis F. Shareck F. (2010). Adaptation of the Highly Productive T7 Expression System to Streptomyces Lividans. Appl. Environ. Microbiol. 76, 967–970. 10.1128/aem.02186-09 20023105 PMC2813025

[B112] Ma Z. Wang P. G. (20191954). RecET Direct Cloning of Polysaccharide Gene Cluster from Gram-Negative Bacteria. Methods Mol. Biol. 1, 15–23. 10.1007/978-1-4939-9154-9_2 30864120

[B113] Makitrynskyy R. Rebets Y. Ostash B. Zaburannyi N. Rabyk M. Walker S. (2010). Genetic Factors that Influence Moenomycin Production in Streptomycetes. J. Ind. Microbiol. Biotechnol. 37, 559–566. 10.1007/s10295-010-0701-1 20204454 PMC2939378

[B114] Makrides S. C. (1996). Strategies for Achieving High-Level Expression of Genes in *Escherichia coli* . Microbiol. Rev. 60, 512–538. 10.1128/mr.60.3.512-538.1996 8840785 PMC239455

[B115] Marti T. Hu Z. Pohl N. L. Shah A. N. Khosla C. (2000). Cloning, Nucleotide Sequence, and Heterologous Expression of the Biosynthetic Gene Cluster for R1128, a Non-steroidal Estrogen Receptor Antagonist. Insights into an Unusual Priming Mechanism. J. Biol. Chem. 275, 33443–33448. 10.1074/jbc.m006766200 10931852

[B116] Martinez A. Kolvek S. J. Yip C. L. Hopke J. Brown K. A. Macneil I. A. (2004). Genetically Modified Bacterial Strains and Novel Bacterial Artificial Chromosome Shuttle Vectors for Constructing Environmental Libraries and Detecting Heterologous Natural Products in Multiple Expression Hosts. Appl. Environ. Microbiol. 70, 2452–2463. 10.1128/aem.70.4.2452-2463.2004 15066844 PMC383137

[B117] Martinez-Burgo Y. Alvarez-Alvarez R. Perez-Redondo R. Liras P. (2014). Heterologous Expression of Streptomyces Clavuligerus ATCC 27064 Cephamycin C Gene Cluster. J. Biotechnol. 186, 21–29. 10.1016/j.jbiotec.2014.06.002 24975573

[B118] Medema M. H. Breitling R. Takano E. (2011). Synthetic Biology in Streptomyces Bacteria. Methods Enzymol. 497, 485–502. 10.1016/b978-0-12-385075-1.00021-4 21601100

[B119] Miller T. W. Chaiet L. Cole D. J. Cole L. J. Flor J. E. Goegelman R. T. (1979). Avermectins, New Family of Potent Anthelmintic Agents: Isolation and Chromatographic Properties. Antimicrob. Agents Chemother. 15, 368–371. 10.1128/aac.15.3.368 464562 PMC352667

[B120] Moore S. J. Lai H. E. Chee S. M. Toh M. Coode S. Chengan K. (2021). A Streptomyces Venezuelae Cell-free Toolkit for Synthetic Biology. ACS Synth. Biol. 10, 402–411. 10.1021/acssynbio.0c00581 33497199 PMC7901020

[B121] Myronovskyi M. Rosenkranzer B. Nadmid S. Pujic P. Normand P. Luzhetskyy A. (2018). Generation of a Cluster-free Streptomyces Albus Chassis Strains for Improved Heterologous Expression of Secondary Metabolite Clusters. Metab. Eng. 49, 316–324. 10.1016/j.ymben.2018.09.004 30196100

[B122] Nah H. J. Park J. Choi S. Kim E. S. (2021). WblA, a Global Regulator of Antibiotic Biosynthesis in Streptomyces. J. Ind. Microbiol. Biotechnol. 48, 1. 10.1093/jimb/kuab007 PMC9113171 33928363

[B123] Nah H. J. Pyeon H. R. Kang S. H. Choi S. S. Kim E. S. (2017). Cloning and Heterologous Expression of a Large-Sized Natural Product Biosynthetic Gene Cluster in Streptomyces Species. Front. Microbiol. 8, 394. 10.3389/fmicb.2017.00394 28360891 PMC5350119

[B124] Nah H. J. Woo M. W. Choi S. S. Kim E. S. (2015). Precise Cloning and Tandem Integration of Large Polyketide Biosynthetic Gene Cluster Using Streptomyces Artificial Chromosome System. Microb. Cel Fact 14, 140. 10.1186/s12934-015-0325-2 PMC4573296 26377404

[B125] Noda S. Ito Y. Shimizu N. Tanaka T. Ogino C. Kondo A. (2010). Over-production of Various Secretory-form Proteins in Streptomyces Lividans. Protein Expr. Purif. 73, 198–202. 10.1016/j.pep.2010.05.011 20546899

[B126] Noda S. Matsumoto T. Tanaka T. Kondo A. (2015). Secretory Production of Tetrameric Native Full-Length Streptavidin with Thermostability Using Streptomyces Lividans as a Host. Microb. Cel Fact 14, 5. 10.1186/s12934-014-0188-y PMC4328045 25582841

[B127] Noda S. Miyazaki T. Tanaka T. Chiaki O. Kondo A. (2013). High-level Production of Mature Active-form Streptomyces Mobaraensis Transglutaminase via Pro-transglutaminase Processing Using Streptomyces Lividans as a Host. Biochem. Eng. J. 74, 76–80. 10.1016/j.bej.2013.02.011

[B128] Noguchi Y. Kashiwagi N. Uzura A. Ogino C. Kondo A. Ikeda H. (2018). Development of a Strictly Regulated Xylose-Induced Expression System in Streptomyces. Microb. Cel Fact 17, 151. 10.1186/s12934-018-0991-y PMC6149001 30241528

[B129] Novakova R. Nunez L. E. Homerova D. Knirschova R. Feckova L. Rezuchova B. (2018). Increased Heterologous Production of the Antitumoral Polyketide Mithramycin A by Engineered Streptomyces Lividans TK24 Strains. Appl. Microbiol. Biotechnol. 102, 857–869. 10.1007/s00253-017-8642-5 29196786

[B130] Ochi K. (2007). From Microbial Differentiation to Ribosome Engineering. Biosci. Biotechnol. Biochem. 71, 1373–1386. 10.1271/bbb.70007 17587668

[B131] Orfanoudaki G. Economou A. (2014). Proteome-wide Subcellular Topologies of *E. coli* Polypeptides Database (STEPdb). Mol. Cel Proteomics 13, 3674–3687. 10.1074/mcp.o114.041137 PMC4256514 25210196

[B132] Park D. Swayambhu G. Pfeifer B. A. (2020). Heterologous Biosynthesis as a Platform for Producing New Generation Natural Products. Curr. Opin. Biotechnol. 66, 123–130. 10.1016/j.copbio.2020.06.014 32784020

[B133] Park S. R. Ahn M. S. Han A. R. Park J. W. Yoon Y. J. (2011). Enhanced Flavonoid Production in Streptomyces Venezuelae via Metabolic Engineering. J. Microbiol. Biotechnol. 21, 1143–1146. 10.4014/jmb.1108.08012 22127124

[B134] Park S. R. Paik J. H. Ahn M. S. Park J. W. Yoon Y. J. (2010). Biosynthesis of Plant-specific Flavones and Flavonols in Streptomyces Venezuelae. J. Microbiol. Biotechnol. 20, 1295–1299. 10.4014/jmb.1005.05038 20890094

[B135] Park S. R. Yoon J. A. Paik J. H. Park J. W. Jung W. S. Ban Y. H. (2009). Engineering of Plant-specific Phenylpropanoids Biosynthesis in Streptomyces Venezuelae. J. Biotechnol. 141, 181–188. 10.1016/j.jbiotec.2009.03.013 19433224

[B136] Parro V. C. Schacht S. Anne J. Mellado R. P. (1999). Four Genes Encoding Different Type I Signal Peptidases Are Organized in a Cluster in Streptomyces Lividans TK21. Microbiology (Reading) 145 (Pt 9), 2255–2263. 10.1099/00221287-145-9-2255 10517578

[B137] Peng Q. Gao G. Lu J. Long Q. Chen X. Zhang F. (2018). Engineered Streptomyces Lividans Strains for Optimal Identification and Expression of Cryptic Biosynthetic Gene Clusters. Front. Microbiol. 9, 3042. 10.3389/fmicb.2018.03042 30619133 PMC6295570

[B138] Pfeifer B. A. Khosla C. (2001). Biosynthesis of Polyketides in Heterologous Hosts. Microbiol. Mol. Biol. Rev. 65, 106–118. 10.1128/mmbr.65.1.106-118.2001 11238987 PMC99020

[B139] Pham V. T. T. Nguyen C. T. Dhakal D. Nguyen H. T. Kim T.-S. Sohng J. K. (2021). Recent Advances in the Heterologous Biosynthesis of Natural Products from Streptomyces. Appl. Sci. 11, 1851. 10.3390/app11041851

[B140] Poelarends G. J. Mazurkiewicz P. Konings W. N. (2002). Multidrug Transporters and Antibiotic Resistance in Lactococcus Lactis. Biochim. Biophys. Acta 1555, 1–7. 10.1016/s0005-2728(02)00246-3 12206883

[B141] Pulido D. Jimenez A. Salas M. Mellado R. P. (1987). A Bacillus Subtilis Phage Phi 29 Transcription Terminator Is Efficiently Recognized in Streptomyces Lividans. Gene 56, 277–282. 10.1016/0378-1119(87)90144-2 2824291

[B142] Pyeon H. R. Nah H. J. Kang S. H. Choi S. S. Kim E. S. (2017). Heterologous Expression of Pikromycin Biosynthetic Gene Cluster Using Streptomyces Artificial Chromosome System. Microb. Cel Fact 16, 96. 10.1186/s12934-017-0708-7 PMC5452415 28569150

[B143] Qian Z. Bruhn T. D'agostino P. M. Herrmann A. Haslbeck M. Antal N. (2020). Discovery of the Streptoketides by Direct Cloning and Rapid Heterologous Expression of a Cryptic PKS II Gene Cluster from Streptomyces Sp. Tu 6314. J. Org. Chem. 85, 664–673. 10.1021/acs.joc.9b02741 31746205

[B144] Rodriguez-Garcia A. Combes P. Perez-Redondo R. Smith M. C. Smith M. C. (2005). Natural and Synthetic Tetracycline-Inducible Promoters for Use in the Antibiotic-Producing Bacteria Streptomyces. Nucleic Acids Res. 33, e87. 10.1093/nar/gni086 15917435 PMC1140374

[B145] Ruckert C. Albersmeier A. Busche T. Jaenicke S. Winkler A. Friethjonsson O. H. (2015). Complete Genome Sequence of Streptomyces Lividans TK24. J. Biotechnol. 199, 21–22. 10.1016/j.jbiotec.2015.02.004 25680930

[B146] Rudolph M. M. Vockenhuber M. P. Suess B. (2013). Synthetic Riboswitches for the Conditional Control of Gene Expression in Streptomyces Coelicolor. Microbiology (Reading) 159, 1416–1422. 10.1099/mic.0.067322-0 23676435

[B147] Seghezzi N. Amar P. Koebmann B. Jensen P. R. Virolle M. J. (2011). The Construction of a Library of Synthetic Promoters Revealed Some Specific Features of strong Streptomyces Promoters. Appl. Microbiol. Biotechnol. 90, 615–623. 10.1007/s00253-010-3018-0 21243353

[B148] Sevillano L. Vijgenboom E. Van Wezel G. P. Diaz M. Santamaria R. I. (2016). New Approaches to Achieve High Level Enzyme Production in Streptomyces Lividans. Microb. Cel Fact 15, 28. 10.1186/s12934-016-0425-7 PMC4743123 26846788

[B149] Shao M. Ma J. Li Q. Ju J. (2019). Identification of the Anti-infective Aborycin Biosynthetic Gene Cluster from Deep-Sea-Derived Streptomyces Sp. SCSIO ZS0098 Enables Production in a Heterologous Host. Mar. Drugs 17. 10.3390/md17020127 PMC6409603 30795576

[B150] Shao Z. Luo Y. Zhao H. (2012). DNA Assembler Method for Construction of Zeaxanthin-Producing Strains of *Saccharomyces cerevisiae* . Methods Mol. Biol. 898, 251–262. 10.1007/978-1-61779-918-1_17 22711131 PMC4323269

[B151] Shao Z. Rao G. Li C. Abil Z. Luo Y. Zhao H. (2013). Refactoring the Silent Spectinabilin Gene Cluster Using a Plug-And-Play Scaffold. ACS Synth. Biol. 2, 662–669. 10.1021/sb400058n 23968564 PMC4326240

[B152] Shao Z. Zhao H. (2013). Construction and Engineering of Large Biochemical Pathways via DNA Assembler. Methods Mol. Biol. 1073, 85–106. 10.1007/978-1-62703-625-2_9 23996442 PMC4321867

[B153] Shao Z. Zhao H. Zhao H. (2009). DNA Assembler, an *In Vivo* Genetic Method for Rapid Construction of Biochemical Pathways. Nucleic Acids Res. 37, e16. 10.1093/nar/gkn991 19074487 PMC2632897

[B154] Sharma V. Kaur R. Salwan R. (2021). Streptomyces: Host for Refactoring of Diverse Bioactive Secondary Metabolites. 3 Biotech. 11, 340. 10.1007/s13205-021-02872-y PMC8209132 34221811

[B155] Shima J. Hesketh A. Okamoto S. Kawamoto S. Ochi K. (1996). Induction of Actinorhodin Production by rpsL (Encoding Ribosomal Protein S12) Mutations that Confer Streptomycin Resistance in Streptomyces Lividans and Streptomyces Coelicolor A3(2). J. Bacteriol. 178, 7276–7284. 10.1128/jb.178.24.7276-7284.1996 8955413 PMC178644

[B156] Siegl T. Tokovenko B. Myronovskyi M. Luzhetskyy A. (2013). Design, Construction and Characterisation of a Synthetic Promoter Library for fine-tuned Gene Expression in Actinomycetes. Metab. Eng. 19, 98–106. 10.1016/j.ymben.2013.07.006 23876413

[B157] Sinsereekul N. Wangkam T. Thamchaipenet A. Srikhirin T. Eurwilaichitr L. Champreda V. (2010). Recombinant Expression of BTA Hydrolase in Streptomyces Rimosus and Catalytic Analysis on Polyesters by Surface Plasmon Resonance. Appl. Microbiol. Biotechnol. 86, 1775–1784. 10.1007/s00253-010-2465-y 20174792

[B158] Skiba M. A. Maloney F. P. Dan Q. Fraley A. E. Aldrich C. C. Smith J. L. (2018). PKS-NRPS Enzymology and Structural Biology: Considerations in Protein Production. Methods Enzymol. 604, 45–88. 10.1016/bs.mie.2018.01.035 29779664 PMC5992914

[B159] Snajder M. Carrillo Rincon A. F. Magdevska V. Bahun M. Kranjc L. Pas M. (2019). Extracellular Production of the Engineered Thermostable Protease Pernisine from Aeropyrum Pernix K1 in Streptomyces Rimosus. Microb. Cel Fact 18, 196. 10.1186/s12934-019-1245-3 PMC6839199 31699090

[B160] Sommer B. Friehs K. Flaschel E. Reck M. Stahl F. Scheper T. (2009). Extracellular Production and Affinity Purification of Recombinant Proteins with *Escherichia coli* Using the Versatility of the Maltose Binding Protein. J. Biotechnol. 140, 194–202. 10.1016/j.jbiotec.2009.01.010 19428714

[B161] Tan G. Y. Deng K. Liu X. Tao H. Chang Y. Chen J. (2017). Heterologous Biosynthesis of Spinosad: An Omics-Guided Large Polyketide Synthase Gene Cluster Reconstitution in Streptomyces. ACS Synth. Biol. 6, 995–1005. 10.1021/acssynbio.6b00330 28264562

[B162] Tang W. Guo Z. Cao Z. Wang M. Li P. Meng X. (2018). d-Sedoheptulose-7-phosphate Is a Common Precursor for the Heptoses of Septacidin and Hygromycin B. Proc. Natl. Acad. Sci. U S A. 115, 2818–2823. 10.1073/pnas.1711665115 29483275 PMC5856511

[B163] Tang X. Li J. Millan-Aguinaga N. Zhang J. J. O'neill E. C. Ugalde J. A. (2015). Identification of Thiotetronic Acid Antibiotic Biosynthetic Pathways by Target-Directed Genome Mining. ACS Chem. Biol. 10, 2841–2849. 10.1021/acschembio.5b00658 26458099 PMC4758359

[B164] Tao W. Chen L. Zhao C. Wu J. Yan D. Deng Z. (2019a). *In Vitro* Packaging Mediated One-step Targeted Cloning of Natural Product Pathway. ACS Synth. Biol. 8, 1991–1997. 10.1021/acssynbio.9b00248 31487454

[B165] Tao X. Zhao M. Zhang Y. Liu M. Liu Q. Wang W. (2019b). Comparison of the Expression of Phospholipase D from Streptomyces Halstedii in Different Hosts and its Over-expression in Streptomyces Lividans. FEMS Microbiol. Lett. 366, fnz051. 10.1093/femsle/fnz051 30869776

[B166] Temuujin U. Chi W. J. Chang Y. K. Hong S. K. (2012). Identification and Biochemical Characterization of Sco3487 from Streptomyces Coelicolor A3(2), an Exo- and Endo-type Beta-Agarase-Producing Neoagarobiose. J. Bacteriol. 194, 142–149. 10.1128/jb.05978-11 22020647 PMC3256618

[B167] Temuujin U. Chi W. J. Lee S. Y. Chang Y. K. Hong S. K. (2011). Overexpression and Biochemical Characterization of DagA from Streptomyces Coelicolor A3(2): an Endo-type Beta-Agarase Producing Neoagarotetraose and Neoagarohexaose. Appl. Microbiol. Biotechnol. 92, 749–759. 10.1007/s00253-011-3347-7 21655986

[B168] Thanapipatsiri A. Claesen J. Gomez-Escribano J. P. Bibb M. Thamchaipenet A. (2015). A Streptomyces Coelicolor Host for the Heterologous Expression of Type III Polyketide Synthase Genes. Microb. Cel Fact 14, 145. 10.1186/s12934-015-0335-0 PMC4573997 26376792

[B169] Thomas J. D. Daniel R. A. Errington J. Robinson C. (2001). Export of Active green Fluorescent Protein to the Periplasm by the Twin-Arginine Translocase (Tat) Pathway in *Escherichia coli* . Mol. Microbiol. 39, 47–53. 10.1046/j.1365-2958.2001.02253.x 11123687

[B170] Torres-Bacete J. Hormigo D. Torres-Guzman R. Arroyo M. Castillon M. P. Garcia L. (2015). Overexpression of Penicillin V Acylase from Streptomyces Lavendulae and Elucidation of its Catalytic Residues. Appl. Environ. Microbiol. 81, 1225–1233. 10.1128/aem.02352-14 25501472 PMC4309716

[B171] Trauger J. W. Walsh C. T. (2000). Heterologous Expression in *Escherichia coli* of the First Module of the Nonribosomal Peptide Synthetase for Chloroeremomycin, a Vancomycin-type Glycopeptide Antibiotic. Proc. Natl. Acad. Sci. U S A. 97, 3112–3117. 10.1073/pnas.97.7.3112 10716695 PMC16201

[B172] Tripathi N. K. Shrivastava A. (2019). Recent Developments in Bioprocessing of Recombinant Proteins: Expression Hosts and Process Development. Front. Bioeng. Biotechnol. 7, 420. 10.3389/fbioe.2019.00420 31921823 PMC6932962

[B173] Tsolis K. C. Tsare E. P. Orfanoudaki G. Busche T. Kanaki K. Ramakrishnan R. (2018). Comprehensive Subcellular Topologies of Polypeptides in Streptomyces. Microb. Cel Fact 17, 43. 10.1186/s12934-018-0892-0 PMC5853079 29544487

[B174] Tu J. Li S. Chen J. Song Y. Fu S. Ju J. (2018). Characterization and Heterologous Expression of the Neoabyssomicin/abyssomicin Biosynthetic Gene Cluster from Streptomyces Koyangensis SCSIO 5802. Microb. Cel Fact 17, 28. 10.1186/s12934-018-0875-1 PMC5819245 29463238

[B175] Van Mellaert L. Mei L. Lammertyn E. Schacht S. Ann J. (1998). Site-specific Integration of Bacteriophage VWB Genome into Streptomyces Venezuelae and Construction of a VWB-Based Integrative Vector. Microbiology (Reading) 144 (Pt 12), 3351–3358. 10.1099/00221287-144-12-3351 9884227

[B176] Van Wezel G. P. Mcdowall K. J. (2011). The Regulation of the Secondary Metabolism of Streptomyces: New Links and Experimental Advances. Nat. Prod. Rep. 28, 1311–1333. 10.1039/c1np00003a 21611665

[B177] Vior N. M. Lacret R. Chandra G. Dorai-Raj S. Trick M. Truman A. W. (2018). Discovery and Biosynthesis of the Antibiotic Bicyclomycin in Distantly Related Bacterial Classes. Appl. Environ. Microbiol. 84. 10.1128/AEM.02828-17 PMC5930311 29500259

[B178] Wang H. Li Z. Jia R. Yin J. Li A. Xia L. (2018). ExoCET: Exonuclease *In Vitro* Assembly Combined with RecET Recombination for Highly Efficient Direct DNA Cloning from Complex Genomes. Nucleic Acids Res. 46, 2697. 10.1093/nar/gkx1296 29272442 PMC5861451

[B179] Wang K. Liu X. F. Bu Q. T. Zheng Y. Chen X. A. Li Y. Q. (2019a). Transcriptome-Based Identification of a Strong Promoter for Hyper-Production of Natamycin in Streptomyces. Curr. Microbiol. 76, 95–99. 10.1007/s00284-018-1589-7 30421143

[B180] Wang W. Li X. Wang J. Xiang S. Feng X. Yang K. (2013). An Engineered strong Promoter for Streptomycetes. Appl. Environ. Microbiol. 79, 4484–4492. 10.1128/aem.00985-13 23686264 PMC3697493

[B181] Wang X. Yin S. Bai J. Liu Y. Fan K. Wang H. (2019b). Heterologous Production of Chlortetracycline in an Industrial Grade Streptomyces Rimosus Host. Appl. Microbiol. Biotechnol. 103, 6645–6655. 10.1007/s00253-019-09970-1 31240365

[B182] Ward J. M. Janssen G. R. Kieser T. Bibb M. J. Buttner M. J. Bibb M. J. (1986). Construction and Characterisation of a Series of Multi-Copy Promoter-Probe Plasmid Vectors for Streptomyces Using the Aminoglycoside Phosphotransferase Gene from Tn5 as Indicator. Mol. Gen. Genet. 203, 468–478. 10.1007/bf00422072 3018431

[B183] Weiner J. H. Bilous P. T. Shaw G. M. Lubitz S. P. Frost L. Thomas G. H. (1998). A Novel and Ubiquitous System for Membrane Targeting and Secretion of Cofactor-Containing Proteins. Cell 93, 93–101. 10.1016/s0092-8674(00)81149-6 9546395

[B184] Wendt-Pienkowski E. Huang Y. Zhang J. Li B. Jiang H. Kwon H. (2005). Cloning, Sequencing, Analysis, and Heterologous Expression of the Fredericamycin Biosynthetic Gene Cluster from Streptomyces Griseus. J. Am. Chem. Soc. 127, 16442–16452. 10.1021/ja054376u 16305230

[B185] Winter J. M. Moffitt M. C. Zazopoulos E. Mcalpine J. B. Dorrestein P. C. Moore B. S. (2007). Molecular Basis for Chloronium-Mediated Meroterpene Cyclization: Cloning, Sequencing, and Heterologous Expression of the Napyradiomycin Biosynthetic Gene Cluster. J. Biol. Chem. 282, 16362–16368. 10.1074/jbc.m611046200 17392281

[B186] Xia H. Li X. Li Z. Zhan X. Mao X. Li Y. (2020). The Application of Regulatory Cascades in Streptomyces: Yield Enhancement and Metabolite Mining. Front. Microbiol. 11, 406. 10.3389/fmicb.2020.00406 32265866 PMC7105598

[B187] Xu M. Wang Y. Zhao Z. Gao G. Huang S. X. Kang Q. (2016). Functional Genome Mining for Metabolites Encoded by Large Gene Clusters through Heterologous Expression of a Whole-Genome Bacterial Artificial Chromosome Library in Streptomyces Spp. Appl. Environ. Microbiol. 82, 5795–5805. 10.1128/aem.01383-16 27451447 PMC5038051

[B188] Xue Y. Sherman D. H. (2001). Biosynthesis and Combinatorial Biosynthesis of Pikromycin-Related Macrolides in Streptomyces Venezuelae. Metab. Eng. 3, 15–26. 10.1006/mben.2000.0167 11162229

[B189] Yamanaka K. Reynolds K. A. Kersten R. D. Ryan K. S. Gonzalez D. J. Nizet V. (2014). Direct Cloning and Refactoring of a Silent Lipopeptide Biosynthetic Gene Cluster Yields the Antibiotic Taromycin A. Proc. Natl. Acad. Sci. U S A. 111, 1957–1962. 10.1073/pnas.1319584111 24449899 PMC3918841

[B190] Yang D. Zhu X. Wu X. Feng Z. Huang L. Shen B. (2011). Titer Improvement of Iso-Migrastatin in Selected Heterologous Streptomyces Hosts and Related Analysis of mRNA Expression by Quantitative RT-PCR. Appl. Microbiol. Biotechnol. 89, 1709–1719. 10.1007/s00253-010-3025-1 21132287 PMC3105603

[B191] Yang T. Yang K. Chen Y. Fan K. (2019). Characterization of a Bi-directional Promoter OtrRp Involved in Oxytetracycline Biosynthesis. Curr. Microbiol. 76, 1264–1269. 10.1007/s00284-019-01753-1 31410507

[B192] Yin J. Hoffmann M. Bian X. Tu Q. Yan F. Xia L. (2015). Direct Cloning and Heterologous Expression of the Salinomycin Biosynthetic Gene Cluster from Streptomyces Albus DSM41398 in Streptomyces Coelicolor A3(2). Sci. Rep. 5, 15081. 10.1038/srep15081 26459865 PMC4602208

[B193] Yin S. Li Z. Wang X. Wang H. Jia X. Ai G. (2016). Heterologous Expression of Oxytetracycline Biosynthetic Gene Cluster in Streptomyces Venezuelae WVR2006 to Improve Production Level and to Alter Fermentation Process. Appl. Microbiol. Biotechnol. 100, 10563–10572. 10.1007/s00253-016-7873-1 27709288

[B194] Zaburannyi N. Rabyk M. Ostash B. Fedorenko V. Luzhetskyy A. (2014). Insights into Naturally Minimised Streptomyces Albus J1074 Genome. BMC Genomics 15, 97. 10.1186/1471-2164-15-97 24495463 PMC3937824

[B195] Zhang J. J. Tang X. Moore B. S. (2019). Genetic Platforms for Heterologous Expression of Microbial Natural Products. Nat. Prod. Rep. 36, 1313–1332. 10.1039/c9np00025a 31197291 PMC6750982

[B196] Zhang J. J. Tang X. Zhang M. Nguyen D. Moore B. S. (2017a). Broad-Host-Range Expression Reveals Native and Host Regulatory Elements that Influence Heterologous Antibiotic Production in Gram-Negative Bacteria. mBio 8. 10.1128/mBio.01291-17 PMC5587914 28874475

[B197] Zhang L. Zhu B. Dai R. Zhao G. Ding X. (2013). Control of Directionality in Streptomyces Phage phiBT1 Integrase-Mediated Site-specific Recombination. PLoS One 8, e80434. 10.1371/journal.pone.0080434 24278283 PMC3836970

[B198] Zhang X. Lu C. Bai L. (2017b). Conversion of the High-Yield Salinomycin Producer Streptomyces Albus BK3-25 into a Surrogate Host for Polyketide Production. Sci. China Life Sci. 60, 1000–1009. 10.1007/s11427-017-9122-8 28812299

[B199] Zhou M. Jing X. Xie P. Chen W. Wang T. Xia H. (2012). Sequential Deletion of All the Polyketide Synthase and Nonribosomal Peptide Synthetase Biosynthetic Gene Clusters and a 900-kb Subtelomeric Sequence of the Linear Chromosome of Streptomyces Coelicolor. FEMS Microbiol. Lett. 333, 169–179. 10.1111/j.1574-6968.2012.02609.x 22670631

[B200] Zhou Y. Murphy A. C. Samborskyy M. Prediger P. Dias L. C. Leadlay P. F. (2015a). Iterative Mechanism of Macrodiolide Formation in the Anticancer Compound Conglobatin. Chem. Biol. 22, 745–754. 10.1016/j.chembiol.2015.05.010 26091168 PMC4504003

[B201] Zhou Z. Xu Q. Bu Q. Guo Y. Liu S. Liu Y. (2015b). Genome Mining-Directed Activation of a Silent Angucycline Biosynthetic Gene Cluster in Streptomyces Chattanoogensis. Chembiochem 16, 496–502. 10.1002/cbic.201402577 25511454

[B202] Zhu Y. Wang L. Du Y. Wang S. Yu T. Hong B. (2011). Heterologous Expression of Human Interleukin-6 in Streptomyces Lividans TK24 Using Novel Secretory Expression Vectors. Biotechnol. Lett. 33, 253–261. 10.1007/s10529-010-0428-0 20931350

[B203] Zimmer R. Verrinder Gibbins A. M. (1997). Construction and Characterization of a Large-Fragment Chicken Bacterial Artificial Chromosome Library. Genomics 42, 217–226. 10.1006/geno.1997.4738 9192841

